# Current and future trends in socio-economic, demographic and governance factors affecting global primate conservation

**DOI:** 10.7717/peerj.9816

**Published:** 2020-08-21

**Authors:** Alejandro Estrada, Paul A. Garber, Abhishek Chaudhary

**Affiliations:** 1 National Autonomous University of Mexico, Institute of Biology, Mexico City, Mexico; 2 Department of Anthropology, Program in Ecology, Evolution, and Conservation Biology, University of Illinois at Urbana-Champaign, Urbana-Champaign, IL, USA; 3 International Centre of Biodiversity and Primate Conservation, Dali, Yunnan, China; 4 Department of Civil Engineering, Indian Institute of Technology, Kanpur, Kanpur, India

**Keywords:** Primates, Biodiversity, Tropical deforestation, Human development, Poverty, Food security, Corruption, Governance, Civil unrest, Human population growth

## Abstract

Currently, ~65% of extant primate species (*ca* 512 species) distributed in 91 countries in the Neotropics, mainland Africa, Madagascar, South Asia and Southeast Asia are threatened with extinction and 75% have declining populations as a result of deforestation and habitat loss resulting from increasing global market demands, and land conversion for industrial agriculture, cattle production and natural resource extraction. Other pressures that negatively impact primates are unsustainable bushmeat hunting, the illegal trade of primates as pets and as body parts, expanding road networks in previously isolated areas, zoonotic disease transmission and climate change. Here we examine current and future trends in several socio-economic factors directly or indirectly affecting primates to further our understanding of the interdependent relationship between human well-being, sustainable development, and primate population persistence. We found that between 2001 and 2018 *ca* 191 Mha of tropical forest (30% canopy cover) were lost as a result of human activities in the five primate range regions. Forty-six percent of this loss was in the Neotropics (Mexico, Central and South America), 30% in Southeast Asia, 21% in mainland Africa, 2% in Madagascar and 1% in South Asia. Countries with the greatest losses (*ca* 57% of total tree cover loss) were Brazil, Indonesia, DRC, China, and Malaysia. Together these countries harbor almost 50% of all extant primate species. In 2018, the world human population was estimated at *ca* 8bn people, *ca* 60% of which were found in primate range countries. Projections to 2050 and to 2100 indicate continued rapid growth of the human populations in these five primate range regions, with Africa surpassing all the other regions and totaling *ca* 4bn people by the year 2100. Socioeconomic indicators show that, compared to developed nations, most primate range countries are characterized by high levels of poverty and income inequality, low human development, low food security, high levels of corruption and weak governance. Models of Shared Socioeconomic Pathway scenarios (SSPs) projected to 2050 and 2100 showed that whereas practices of increasing inequality (SSP4) or unconstrained growth in economic output and energy use (SSP5) are projected to have dire consequences for human well-being and primate survivorship, practices of sustainability-focused growth and equality (SSP1) are expected to have a positive effect on maintaining biodiversity, protecting environments, and improving the human condition. These results stress that improving the well-being, health, and security of the current and future human populations in primate range countries are of paramount importance if we are to move forward with effective policies to protect the world’s primate species and promote biodiversity conservation.

## Introduction

**Figure 1:**
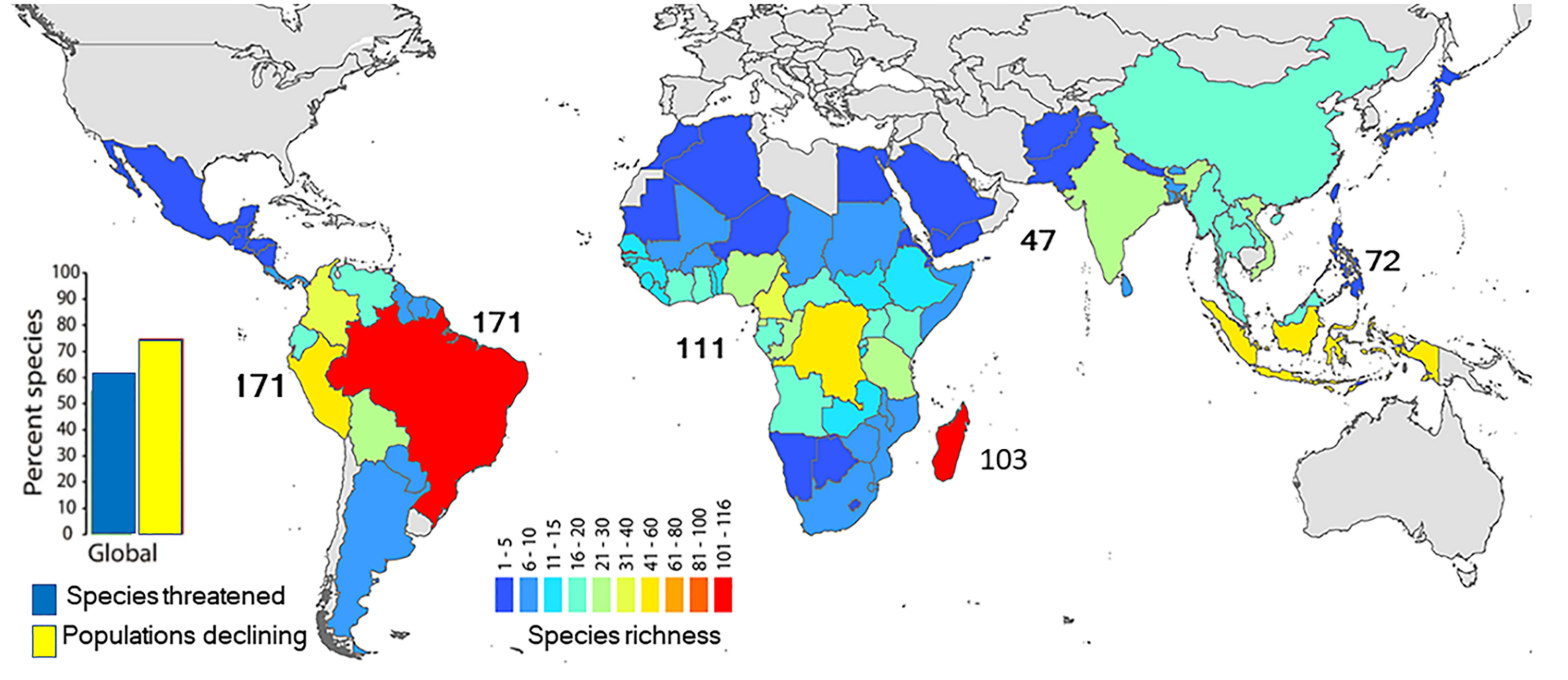
Geographic distribution of primate species richness. Species richness in the main regions where primates are naturally found: the Neotropics, mainland Africa (includes small associated islands), Madagascar, South Asia, and Southeast Asia. The country colors indicate the number of primate species in each country. Number by each region indicates the regional number of primate species. Madagascar stands out with its rich and endemic lemur fauna. Black dots in open spaces between continents are small islands. Source of information: (Estrada et al., 2017; IUCN Red List, 2019). Raw data in Table S1.

A growing global human population and expanding economic activities are exerting unsustainable demands on natural resource extraction and the conversion of forested land for industrial agriculture, cattle production, and the expansion of urban centers (Rockström et al., 2009; Crist, Mora & Engelman, 2017; Crist, 2018; https://populationmatters.org/the-issue). These pressures are negatively impacting critical global commons needed to sustain life on Earth through air, water, and soil pollution, carbon emissions, deforestation and the critical loss of biodiversity (Rockström et al., 2009; Otero et al., 2020; UNGEF, 2020). For example, the atmosphere is being degraded by increased greenhouse gas emissions, ocean acidification is increasing, and tropical forests are being cut at a rate of *ca* 10 million ha per year (Rockström et al., 2009; Steffen et al., 2015; GFW, 2019). Regrettably, recent estimates indicate that some one million animal and plant species are threatened with extinction as a result of human activities (IPBES, 2019). This includes many of the world’s non-human primates, our closest living biological relatives. Primates represent the third most speciose mammalian radiation (*ca* 512 species; Estrada et al., 2017) after bats and rodents. They (prosimians, tarsiers, monkeys and apes) are distributed across 91 countries, principally in the Neotropics, mainland Africa, Madagascar, South Asia and Southeast Asia, and are a critical part of our planet’s biodiversity (Fig. 1; Table S1). Primates are essential models for understanding human evolution, behavior and health (Phillips et al., 2014). The activities of lemur, lorises, tarsiers, monkeys and apes sustain a range of community-wide ecological functions and services (e.g., seed dispersal, pollination, predator-prey relationships) in tropical and temperate forests, that also benefit local human communities (Estrada et al., 2017). Alarmingly, 65% of primate species are threatened with extinction and 75% have declining populations as a result of relentless human pressures on natural environments leading to widespread loss and degradation of tropical forests (Estrada et al., 2017; IUCN Red List, 2019) (Table S1).

A major driver of primate population decline is land use changes driven by global market demands. This has resulted in the widespread loss of primate habitats in order to expand agricultural and cattle production, and the extraction of natural resources (e.g., minerals, oil, wood) to feed and sustain a growing global human population (now *ca* 8 billion set to approach *ca* 10 billion by 2050; https://www.un.org/en/sections/issues-depth/population/) and a rapidly expanding global middle-class diet that emphasizes greater meat consumption (UNGEF, 2019). Hunting for bushmeat, the illegal primate trade, infectious disease transmission among humans-domesticated animals-wild animals, and the spread of invasive species constitute important additional pressures that negatively impact primate populations (IUCN Red List, 2019; Estrada et al., 2017; Lappin et al., 2020).

Recent evidence suggests that the human population has expanded beyond the Earth’s sustainable means and that we are using resources faster than our planet can regenerate, with devastating consequences (Steffen et al., 2015). It took humanity *ca* 200,000 years to go from an estimated population size of a few hundred thousand to a population size of one billion, and just 200 years to go from one billion to a population size of almost eight billion (Population Matters, 2019). At present we are adding close to 80 million people each year, as the world moves towards a human population that may exceed 11 billion by 2100 (UNDESA, 2019a).

While a growing global human population and activities associated with land conversion have historically been the major factors contributing to global biodiversity loss via deforestation, habitat degradation and resource extraction activities (IPBES, 2019), it is clear that initiatives solely designed to limit human population growth as a means to solve immediate environmental problems are largely unrealistic (Bradshaw & Brook, 2014). For example, Brazil, a country suffering significant deforestation in past decades has one of the lowest population growth rates among the countries that harbor primates. Also, extensive deforestation in Brazil has occurred in both highly populated areas (i.e., the Atlantic Forest, the Cerrado), as well as in areas of extremely low population density such as the Amazon. The reasons for deforestation and consequent biodiversity impacts in Brazil and other countries represent a combination of historical, economic, political and social factors. Here we use the I = PAT framework, which examines the environmental impact (I) as a function of population (P), affluence (A) and technology (T) (see for example, Dietz & Rosa, 1997).

To further our understanding of the interdependent relationship between human well-being and primate population persistence, we examine current and future trends in several socio-economic, ecological and demographic indicators in countries within primate range regions. These are: tropical forest loss, human population, GDP per capita, human development index (HDI), extreme poverty, food security, corruption, governance quality and civil unrest/conflict status. We acknowledge that our review of these indicators is by no means complete and primate conservation requires global action to limit both the long-term environmental and economic footprint of the world’s human population, and the overconsumption of natural resources from the tropics by citizens in a small number of developed nations (Estrada, Garber & Chaudhary, 2019). Finally, by focusing on the level of primate regions rather than individual countries, our manuscript emphasizes broad patterns rather than country-specific historical, demographic, cultural, and religious factors that affect primate survivorship. It is in light of these considerations that we present our evaluation.

## Survey Methodology

The information we present is based on a review of the literature and analysis of information from several open access databases. These latter sources are listed below. Our analysis of these databases included information on each of the 91 countries that harbor wild primate populations. For purposes of this evaluation we consider the following regions as discrete entities due to their unique primate fauna: the Neotropics (Mexico, Central and South American tropics), mainland Africa (including small islands off its Atlantic coast), Madagascar, South Asia and Southeast Asia. Because Madagascar harbors a nonoverlapping set of the world’s nonhuman primate species (i.e., some 100 endemic lemur species), the result of over 100 million years of isolation from the African continent, it merits separate evaluation. Information on the conservation status of living primates is from Estrada et al., 2017 and from the International Union for the Conservation of Nature https://www.iucnredlist.org/ (IUCN Red List, 2019).

We report human population growth from 1960 to 2018 in each primate range country (*n* = 91) based on information from the World Bank (https://data.worldbank.org/indicator/SP.POP.TOTL) and from the UN Population Division (https://population.un.org/wpp/Download/Standard/Population). Human population growth projections for 2050 and 2100 were obtained from the UN Population Division (https://ourworldindata.org/grapher/un-population-projection-medium-variant). Additional information on population growth trends was obtained from the UN e-Handbook of Statistics (https://stats.unctad.org/handbook/Population/Total.html).

We describe the extent of tropical forest loss in primate range countries from 2001 to 2018 using data from the open access Global Forest Watch database (http://www.globalforestwatch.org/). These estimates are based on remote sensing procedures.

We use the Gross Domestic Product Per Capita (GDPPC) for the period from 1960 to 2018 as an indicator of economic progress in each primate range country. This metric was obtained from the World Bank socioeconomic indicators (https://data.worldbank.org/indicator/NY.GDP.PCAP.CD). We are aware of the limitations of using the GDPPC as a measure of economic development. This indicator is an average, and consequently overlooks the distribution of incomes across each country. In this regard, the GDPPC of a country may be high or above that of another country, however, most of that wealth could be concentrated in a relatively small number of individuals or families, with the overwhelming majority of citizens extremely poor. Examples of high levels of income inequality in primate range nations include China, Brazil and DRC (Estrada et al., 2018).

We used information from the International Institute for Applied Systems Analysis (IIASA) SSPd database (https://tntcat.iiasa.ac.at/SspDb/dsd?Action=htmlpage&page=about) (Table S11) to model future projections of GDPPC for all primate hosting countries under different Shared Socioeconomic Pathways scenarios (SSPs).

Data used to examine human development in primate range countries (1990–2018) were from the UN HDI, which represents a combined measure of life expectancy, school enrollment, literacy, and income. HDI ranges from 0 (lowest) to 1 (highest) (http://worldpopulationreview.com/countries/hdi-by-country/).

We used the 2015 World Bank percent of the population living on <US $1.90 a day as an indicator of extreme poverty in primate range regions. Data were sourced from the World Bank and include information for Europe and North America (US and Canada) and the world average (https://data.worldbank.org/indicator/SI.POV.DDAY).

As a complementary indicator of poverty, we used the 2018 under-five mortality rate in primate range countries. Data were harvested from the World Bank (https://data.worldbank.org/indicator/SH.DTH.MORT).

We assessed food security in primate range regions by using the internationally accepted standard, the Global Food Security Index (GFSI) of The Economist Intelligence Unit Limited (https://foodsecurityindex.eiu.com/Index). The index integrates the core issues of affordability, availability, quality and safety into a quantitative and qualitative model that evaluates these drivers of food security in developing and developed countries. The index also considers a country’s exposure to the impacts of a changing climate; its susceptibility to natural resource risks; and how the country is adapting to these risks. The GFSI is based on data from 113 countries and ranges from zero (lowest food security) to 100 (highest food security) (https://foodsecurityindex.eiu.com/Index).

To document patterns of corruption, we gathered information for each primate range country from the 2019 Corruption Perceptions Index (CPI) of Transparency International (https://www.transparency.org).

We evaluated governance quality in primate range regions for 2016 using the values of four indicators of governance quality (political stability and absence of violence/terrorism, government effectiveness, rule of law, and control of corruption) provided by the World Bank (http://info.worldbank.org/governance/wgi/index.aspx#reports). We compared these values to the average values of 35 high-income countries.

We examined civil conflict in primate range countries by using the 2019 Global Peace Index (GPI) of the Institute for Economics and Peace (https://economicsandpeace.org/) as an indicator of the level of civil conflict for countries in primate range regions. The GPI ranks 163 independent states and territories according to their level of peacefulness. The GPI is the world’s leading measure of global peacefulness. The GPI covers 99.7 per cent of the world’s population, using 23 qualitative and quantitative indicators, and measures the state of peace using three thematic domains: the level of Societal Safety and Security; the extent of Ongoing Domestic and International Conflict; and the degree of Militarization.

Future projections of population parameters under five Shared Socio-Economic Pathway Scenarios (SSPs) from 2010 to 2100 for countries in each primate region were obtained from the IIASA SSP database (https://tntcat.iiasa.ac.at/SspDb/dsd?Action=htmlpage&page=about). These scenarios define future socioeconomic tendencies that encompass a variety of likely effects and comprise: a world of sustainability and fairness (SSPs1); a business as usual scenario following historical trends (SSPs2); a divided world of growing nationalism (SSPs3); a world of continually and growing inequality (SSPs4); and a world of unrestrained economic growth with high levels of energy use (SSPs5) (Riahi et al., 2017). For a discussion of limitations of the SSP scenarios see Chen et al. (2020) and Shuhui & Xuefeng (2019).

We acknowledge that the information contained in these large datasets may differ in their level of trustworthiness and impartiality. For example, some publicly accessible data by international agencies such as the United Nations Food and Agriculture Organization (FAO) denote general estimates. Likewise, data from the IUCN Red List on the population, distribution, and conservation standing of cryptic, rare, or highly inaccessible primate species may be incomplete. In other instances, however, the data were acquired through careful examination by an impartial organization (e.g., UN Population Division, Transparency International) or were objectively certified using remote sensing (e.g., Global Forest Watch). Each of the organizations we used as sources of data indicate in their portals the constraints of the information presented.

## Results

### Tropical forest loss

**Figure 2:**
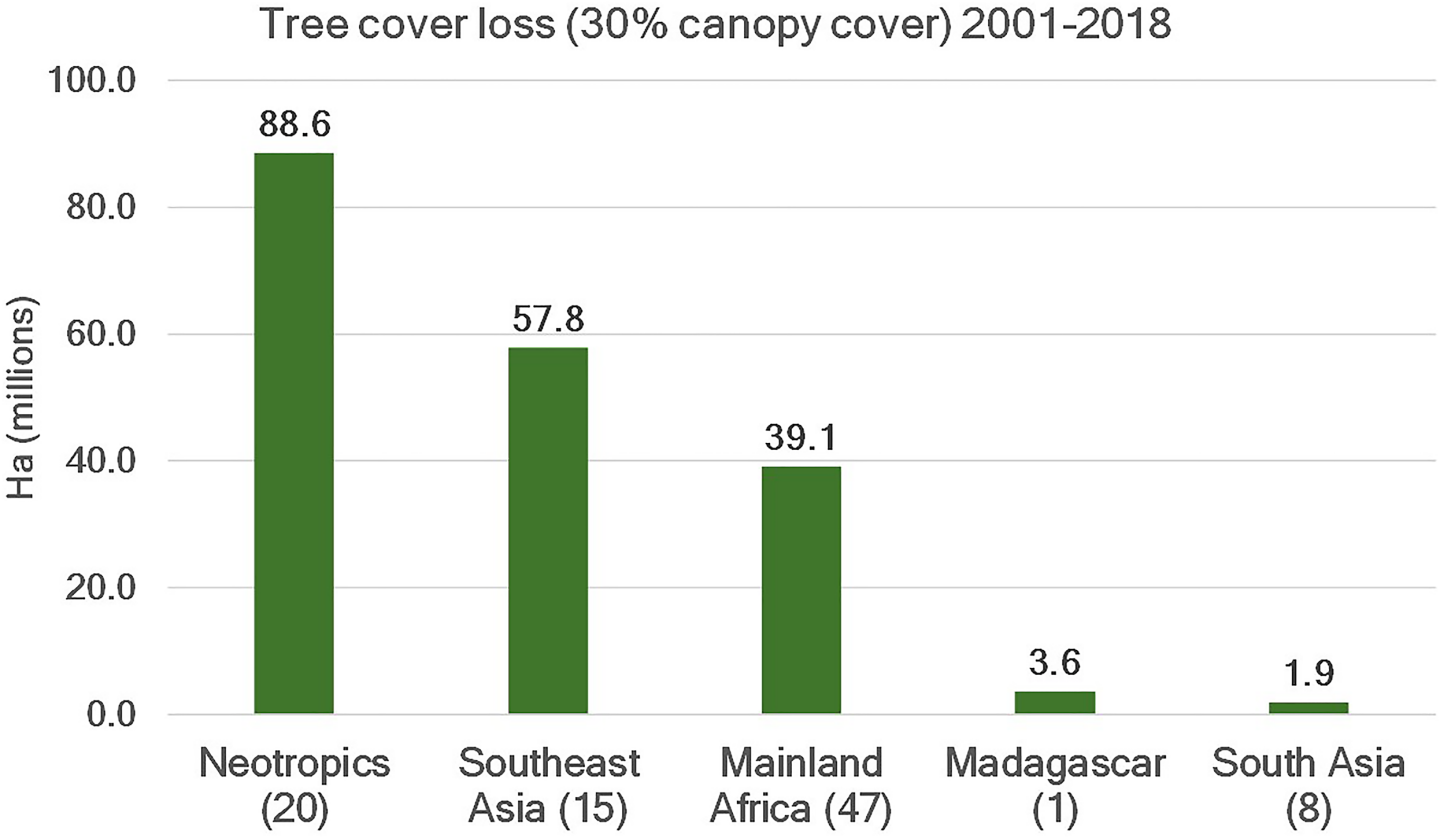
Tree cover loss. Trends in tree cover loss (>30% canopy cover) in primate range regions for the period 2001–2018. Data are from Global Forest Watch (http://www.globalforestwatch.org; accessed February 2020). The number in parentheses on the *x*-axis indicates the number of countries per region included in the analysis. See Table S2 for country data.

**Figure 3:**
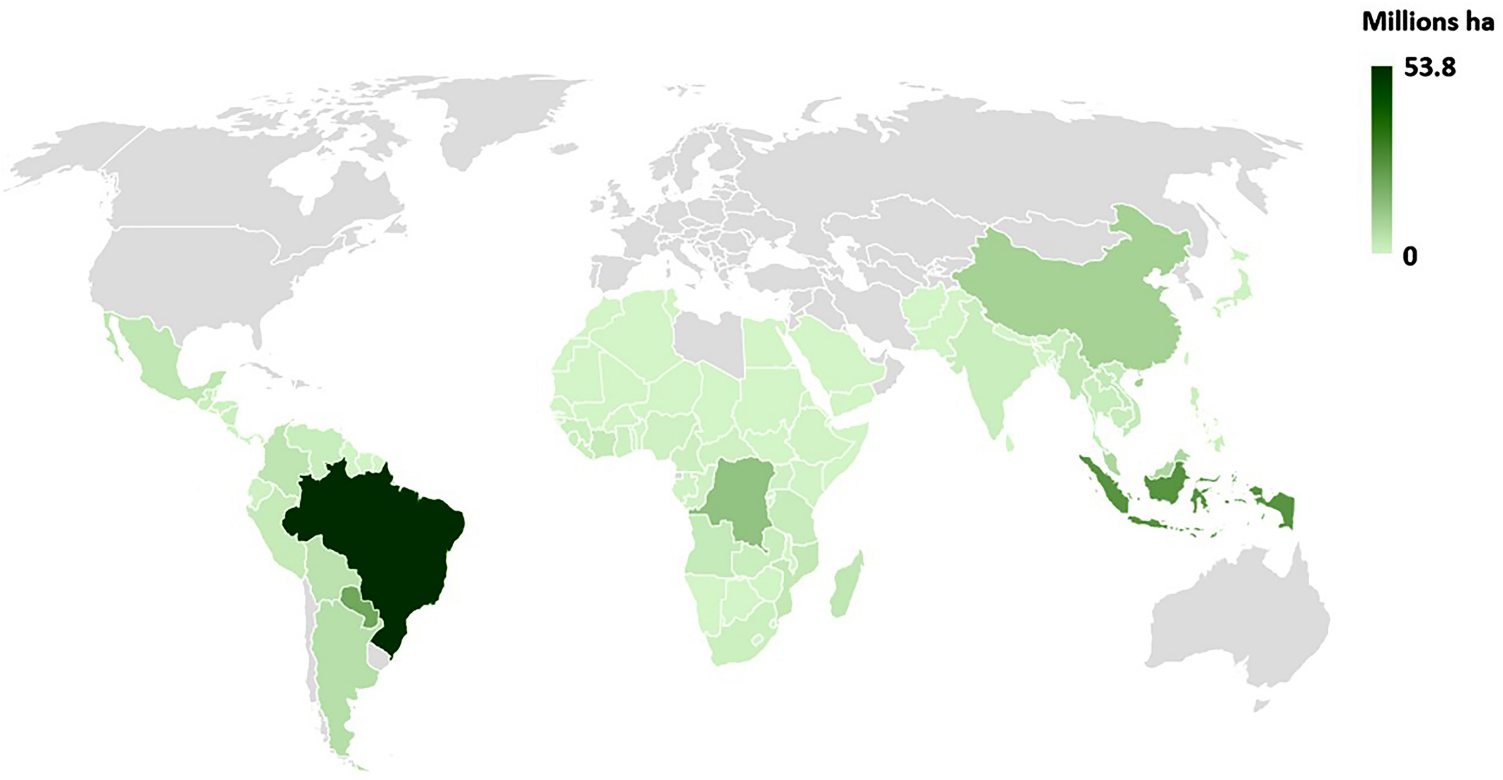
Geographic dstribution of tree cover loss in primate range countries. Map showing the distribution of tree cover loss (>30% canopy cover) in primate range countries for the period 2001–2018. Data are from Global Forest Watch (http://www.globalforestwatch.org; accessed March 2020). No data are shown for nonprimate range countries (in gray). See Table S2 for country data.

Habitat loss is a major driver of the local extinction of primate species. Information from Global Forest Watch (GFW, 2019) indicates that 191 Mha of tropical forest (defined as >30% canopy cover) were lost between 2001 and 2018 as a result of human activities in the five primate range regions (Figs. 2 and 3). This represents an area equivalent to *ca* 43% of the EU land mass. Forty six percent of this loss was in the Neotropics followed by Southeast Asia (30%), mainland Africa (21%), Madagascar (2%) and South Asia (1%). (Figs. 2 and 3; Table S2). Madagascar is home to more than 100 species of lemurs (second richest primate country after Brazil), and over 93% of lemur species are threatened with extinction. Current estimates indicate that Madagascar has lost some 90% of its original forest (Estrada et al., 2018).

Countries with the highest losses of tree cover between 2001 and 2018 were Brazil (53.8 Mha), Indonesia (25.6 Mha), DRC (13.4 Mha), China (9.4 Mha) and Malaysia (7.7 Mha). These five countries totaled 109.9 Mha or *ca* 57% of total tree cover loss (191 Mha) in primate range regions (Fig. S1). Together these countries harbor about 50% of extant primate species. A region by region examination indicates that between 2001 and 2018, the Neotropics lost 88.6 Mha of forest (Fig. S1A), with Brazil, the primate richest country in the world (120 species) accounting for 60% (53.8Mha). During the same period, Indonesia, the third primate richest country accounted for 44% of southeast Asian forest loss (Fig. S1B). An estimated 83% of Indonesian primate species are threatened with extinction. Countries such as China, Malaysia, Myanmar, Lao, Vietnam, Cambodia and Thailand, accounted for an additional 52% of forest loss. Each of these countries has a high proportion of primate species listed as Vulnerable, Endangered or Critically Endangered (Table S1). We note that in other countries, for example, Madagascar, so much native forest has already been cut, that even small amounts of new forest loss will have a dramatic impact on primate survivorship.

The primary drivers of continued forest loss in the five primate range regions include subsistence and industrial agriculture, commodity-driven deforestation, and the expansion of urban areas (Estrada et al., 2017, 2018; Chaudhary & Kastner, 2016; Chaudhary & Brooks, 2019; Estrada, Garber & Chaudhary, 2019). This is unfolding in the context of expanding global economic activities driven principally by the exploitative practices of a small set of multinational corporations and the over-consumption of citizens in a small number of consumer nations who are disproportionately contributing to climate change, pollution, food insecurity, habitat destruction, and income inequality worldwide (Estrada, Garber & Chaudhary, 2019; Chaudhary & Kastner, 2016; Chaudhary & Brooks, 2018, 2019).

### Human population growth in primate range regions

**Figure 4:**
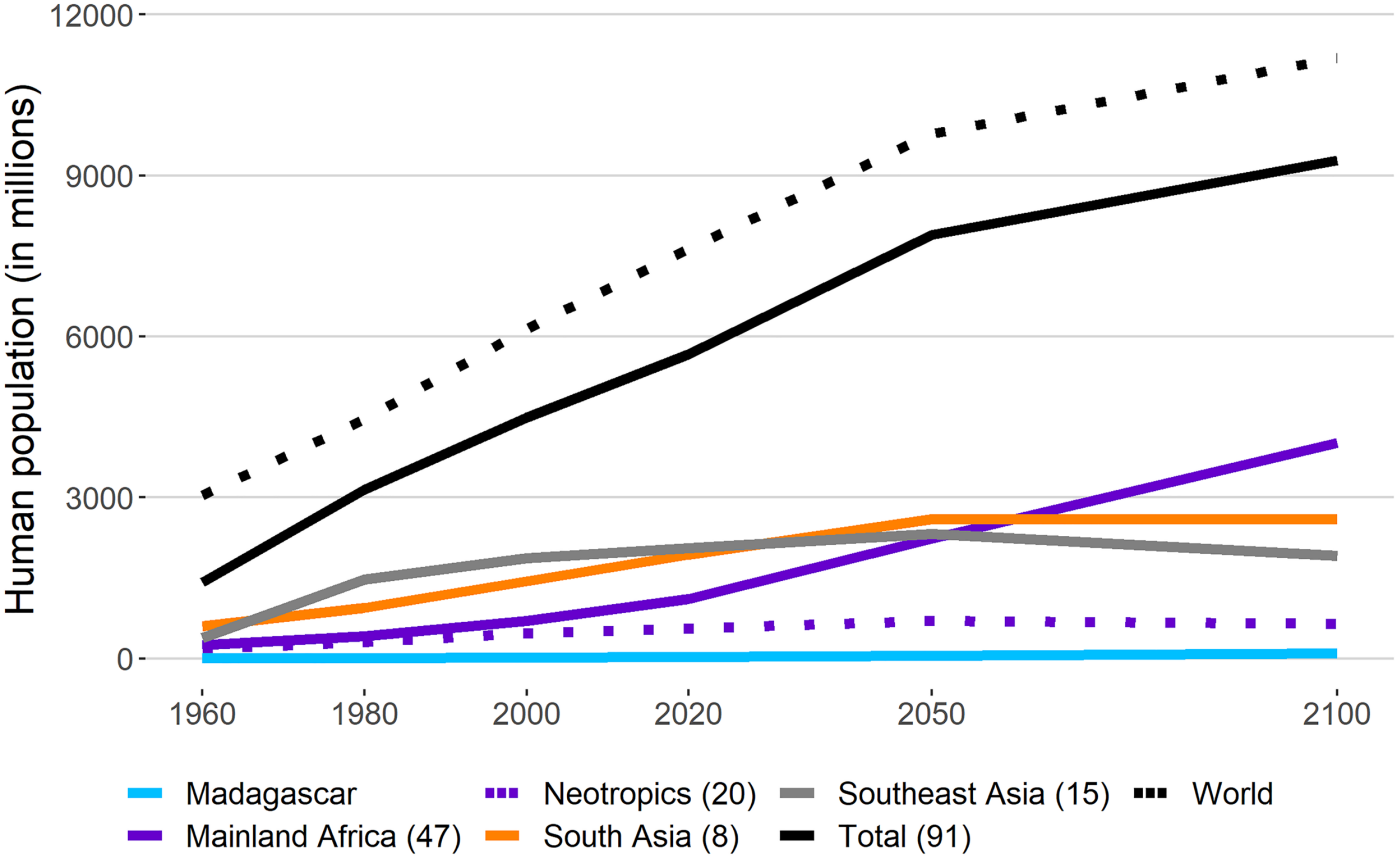
Human population growth in primate range regions. Human population growth in primate range regions between 1960 and 2020 and projections to 2050 and 2100. Also included is the trend for the total world population. Number in parentheses next to each region indicates the number of primate range countries per region. Sources: World Bank https://data.worldbank.org/indicator/SP.POP.TOTL; UN Population Division https://population.un.org/wpp/Download/Standard/Population/; UN Population Prospects 2019 https://ourworldindata.org/grapher/un-population-projection-medium-variant. See Table S3 for country data.

The world´s human population has grown exponentially over the past several decades and a significant segment of this growth has occurred in primate regions (Fig. 4). In 1960 there were 1.4 bn people in primate habitat countries and this number reached 5.6 bn by 2018. Currently human populations in primate range regions account for 74% of the world’s population. United Nations’ population division estimates predict that fully 80% of the world’s population by 2050 (total human population estimated at 9.8 bn) and 83% by 2100 (total human population estimated at 11.2 bn) will reside in primate habitat countries (Fig. 4). Significant human population growth is expected in all primate regions up to 2050, after which Africa will surpass the other four regions with accelerated human population growth continuing to the year 2100 (Fig. 4). Populations in South Asia and Southeast Asia are expected to exhibit reduced growth after 2050. Populations in the Neotropics and Madagascar will grow exponentially, but in the Neotropics growth is expected to decrease slightly after 2050 (Fig. 4).

Demographic models indicate that primate range countries, and especially those with developing economies, are driving the world’s population growth. The population of Africa is increasing at a particularly rapid rate. In 2018, the population growth rate in Africa averaged 2.5%, which was more than double the world average (1.08%) (UNDESA, 2019a; UNCTAD, 2019). Several central and east-central African countries, such as Niger, Uganda, Equatorial Guinea, Angola and the Democratic Republic of the Congo recorded growth rates well above 3 per cent. Several of these countries are among the most primate species-rich in Africa. Rates slightly above the world average were found in South and Southeast Asia (1.2%), with the Neotropics (1.0%) below the world average (World Bank, 2019).

#### Population in rural and urban areas

**Figure 5:**
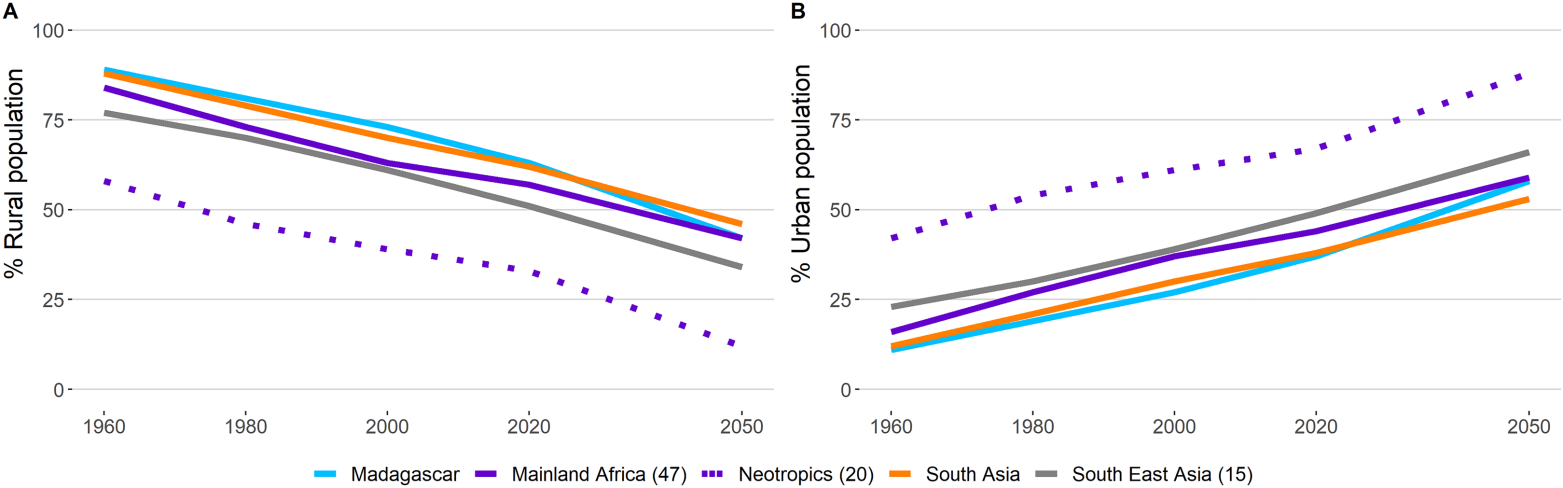
Human population in rural and urban areas in primate range regions. Percentage of the human population living in (A) rural and (B) urban areas in primate range regions. Source: United Nations, Department of Economic and Social Affairs, Population Division. World Urbanization Prospects: The 2018 Revision, Online Edition. Available at https://population.un.org/wup/Download/; Our World in Data. Oxford University. Available at https://ourworldindata.org/grapher/urban-and-rural-population-2050?country=MDG Consulted March 2020. See Table S4 for country data.

Significantly, population growth in primate range regions is expected to occur in urban areas with populations in rural areas declining dramatically (Fig. 5). A sizeable segment of the population in the Neotropics (88%) and Southeast Asia (66%) will reside in urban areas by 2050. The values for mainland Africa are 59%, for Madagascar 53%, and for South Asia also 53% (Fig. 5). While more than half of the increase in the urban population in primate harboring countries is caused by in situ population increase, migration from rural areas into cities also is a contributing factor (Hecht et al., 2015).

While cities cover <2% of the earth’s surface, they use about 78% of the energy produced, including large quantities of nonfood and food products, much of which is wasted, adding to environmental pressures locally and globally (Ulgiati & Zacaro, 2019). Urbanization produces land-cover changes (Grimm et al., 2008) that drive habitat loss, air and water pollution, and the extinction of local animal and plant populations (Hahs et al., 2009). Urban growth entails the movement of goods and services into and out of cities, necessitating the construction of extensive road and rail networks, and the conversion of nearby forests for purposes of agricultural production and industry (Seto, Kaufmann & Woodcock, 2000; Seto et al., 2011). A study of 41 countries in the humid tropics found that forest loss was positively associated with both urban population growth and the export of agricultural products (e.g., cattle, crops) and non-food crops (e.g., palm oil, corn and sugar cane for biofuels), much of which were sold to international markets (DeFries et al., 2010). As cities grow, so do job opportunities. However, many of the new immigrants to large urban centers are the rural poor, who are under-educated and only qualify for low paying jobs in agriculture, manufacturing, and the service sector (Sánchez-Triana et al., 2013). Moreover, there is evidence indicating that urban expansion near protected areas pose important conservation challenges to biodiversity, as protected areas become islands or isolated refuges with limited buffer zones and barriers to animal migration and gene flow (Seto et al., 2011).

The continued growth and expansion of urban areas is expected to exacerbate the negative consequences that deforestation and habitat fragmentation, bushmeat hunting, and capture for the local, regional and international pet trade will have on primate population decline. Given recent assessments that the capture, killing, and trade of wild animals in urban “wet markets” likely represents the source point for the COVID-19 pandemic (Shereen et al., 2020), it is clear that establishing and enforcing laws that promote reforestation in order to expand the physical separation between urban areas and wildlife refuges, and the strict control of unsustainable hunting and live animal capture, are essential components of a successful urban planning strategy. Such a strategy also must include the development of new technologies to increase agricultural yields on non-forested lands and to reduce food waste to more efficiently satisfy the demands for agricultural products in urban areas (DeFries et al., 2010; Seto et al., 2011).

Additional forest loss has resulted from the expansion of dams and mega dams to supply water and electricity to urban areas and nearby industries and to agricultural land (Laurance et al., 2014; Benchimol & Peres, 2015). This has been accompanied by expansion of road and rail networks to transport people, goods and services to resident human populations in urban areas (Alamgir et al., 2017; Clancy, 2008; Laurance, 2006; UNSTATS, 2019). Moreover, the establishment of irregular or unauthorized settlements in the vicinity of cities is an additional factor adding to the cities’ footprint, increasing human-primate conflict and primate population decline (Boyle, 2008).

Although cities harbor poverty, they also are places of innovation and knowledge, and can offer economic opportunities in terms of employment and improved living and health conditions (https://sustainabledevelopment.un.org/topics/sustainablecities). Cities also offer spaces for social and political involvement and the fusion of cultures, reasons why city life is more desirable to some people, especially young adults. For example, a study of 27 countries in Sub-Saharan Africa demonstrated that for the average child under five years of age, living in a rural area and isolated from basic health services and adequate sanitation, the mortality rate (deaths per 1,000 live births) was 49.2% compared to 21.8% for children living in urban areas (Issaka, Agho & Renzaho, 2017).

Unfortunately, rapid urbanization in primate range countries has resulted in a growing number of slum dwellers, a reduction in food security, and inadequate and overburdened infrastructure and services (such as waste collection and water and sanitation systems, roads and transport), leading to unhealthy levels of air and water pollution and unplanned urban sprawl (UNSTATS, 2019). Crowding also can have a devastating effect on disease transmission, especially in developing nations (Hotez, 2017). We found that the proportion of the urban population living in slums is high in primate harboring nations when compared with developed nations. In Madagascar, 77% of the urban population lives in slums, in Sub-Saharan Africa 55%, in South Asia 30%, in Southeast Asia 26% and 20% in the Neotropics. In contrast, in Canada, the United States, and the EU, the percentage of the population living in slums is less than 1% (World Bank, 2020a). While cities and metropolitan areas are centers of economic growth—contributing about 60 per cent of global GDP, they also account for some 70 per cent of global carbon emissions and over 60 per cent of resource use (Li et al., 2019; UNSTATS, 2019). Policy decisions and urban planners need to prioritize reducing the ecological footprint of city residents and industry in order to promote ‘green’ cities, reduce the urban footprint on biodiversity, and actively engage in programs designed to restore native forests (Butler & Laurance, 2008).

Unsurprisingly, the increase in human population size means that more food, water, energy, living space and public health services are required together with additional housing, transportation hubs and networks, road building, and other essential services (Crist, Mora & Engelman, 2017). At the same time, while the human population in developed economies is not growing as rapidly, their economies continue to expand in order to sustain and increase the quality of life of their citizens (UNDESA, 2019a). This has important consequences for biodiversity conservation because these developed nations are characterized by overconsumption and excessive waste (Neff, Spiker & Truant, 2015) of agricultural and nonagricultural commodities, much of which are produced in primate range countries (Estrada, Garber & Chaudhary, 2019). This pattern of “colonial” exploitation, that is, the over-extraction of resources, taking advantage of cheap labor, and environmental degradation, has significantly taxed the natural resources of most primate range nations, and resulted in food insecurity as food is grown to support global supply chains rather than for local consumption (Chaudhary & Kastner, 2016; Chaudhary & Mooers, 2018).

### Socioeconomic indicators and human development

#### GDPPC in primate range countries

**Figure 6:**
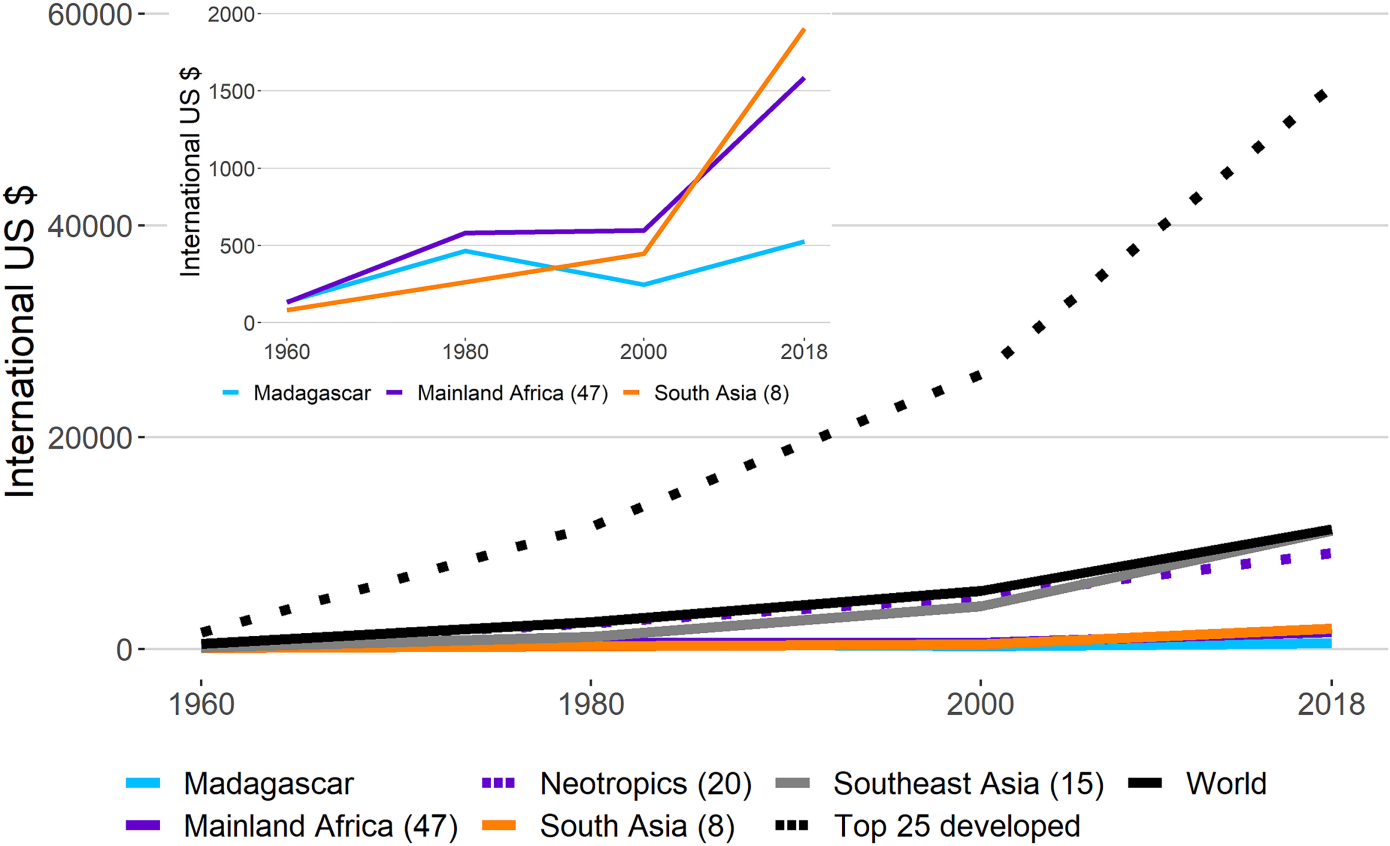
Gross Domestic Product per Capita (GDPPC) in regions harboring primates. Trends in the average values of Gross Domestic Product per Capita (GDPPC) between 1960 and 2018 in primate range regions, the 25 most developed nations, and the world. Data from: World Bank https://data.worldbank.org/indicator/NY.GDP.PCAP.CD. Consulted March 2020. The upper smaller graph is an amplification of trends in mainland Africa, Madagascar and South Asia. Values in parentheses indicate the number of countries with data per region. See Table S5 for country data.

Successful and longstanding primate conservation requires economic resources, satisfactory conservation strategies, efficient implementation of environmental law, public interest, and the practice of sustained and long-term conservation-oriented research. However, if poverty and income inequality are prevalent in the population, primate conservation will not be a primary social concern. Information on the growth of GDPPC between 1960 and 2018 indicates an enormous gap between countries in primate range regions and the top 25 developed nations of the world (Fig. 6; Table S5). And although there has been a gradual increase in the average GDPPC between 1960 and 2018 in countries in primate range regions, only the Neotropics and Southeast Asia have experienced gains that closely match the world average (Fig. 6). In contrast, in mainland Africa, Madagascar and South Asia the level of GDPPC has remained stagnant since 1960, with a small increase between 2000 and 2018 (Fig. 6; Table S5), probably the result of increased exports of agricultural and nonagricultural commodities (Estrada, Garber & Chaudhary, 2019). Yet, in many cases gains in GDPPC in primate range countries such as Brazil, Argentina, Indonesia, Malaysia and China have occurred at the cost of severe and on-going environmental degradation (Table S2; Estrada et al., 2018; Estrada, Garber & Chaudhary, 2019). In general, weak per capita income growth is anticipated over the next few years in mainland Africa, Madagascar and South Asia, regions that harbor a significant part of the global population living in extreme poverty (UNDESA, 2019b). The steep growth curve in the GDPPC for the top 25 developed nations in the world is notable, indicating significant improvements in the standard of living of their populations, and significant differences in the quality of life between citizens in primate range nations and citizens in developed nations (Fig. 6).

Data on the GDPPC for all primate countries at a decadal interval (2010–2100) under five different shared socioeconomic pathway models (SSP-1 to SSP-5) (Samir & Lutz, 2017) show that the highest increase in GDPPC is forecasted under the SSP-5 scenario, which assumes rapid and unconstrained growth in economic output, energy use, and environmental degradation. However, relatively high GDPPC for primate range countries also is predicted under the SSP-1 scenario, which is based on sustainability-focused growth and greater income equality (see Table S11). Similar to projections of population growth, the SSP-1 scenario is a win-win for human development and primate conservation because the growth in GDPPC under this scenario minimizes tropical deforestation and biodiversity loss (Van Vuuren et al., 2017; Estrada, Garber & Chaudhary, 2019).

#### The HDI

**Figure 7:**
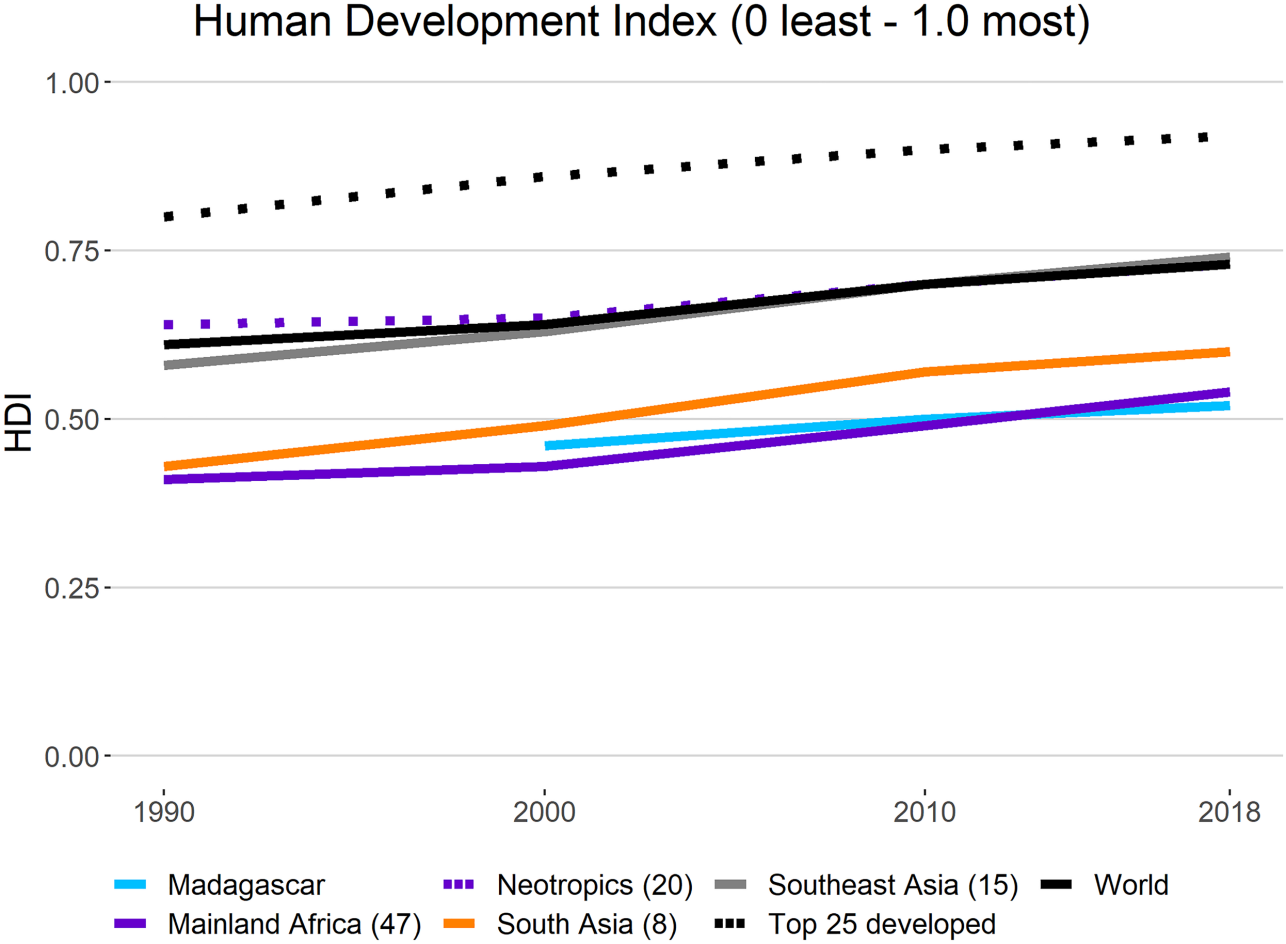
Human Development Index (HDI) in primate range regions. The 1990–2019 Human Development Index (HDI) in primate range regions. (Lowest human development = 0; highest = 1.0). Average values are shown for countries in each region. Also shown is the average HDI for the world and for the top 25 most developed nations. No data are available for Madagascar for 1990. Source: United Nations Development Program (http://hdr.undp.org/en/content/human-development-index-hdi; http://worldpopulationreview.com/countries/hdi-by-country/ accessed March 2020). Values in parentheses represent the number of countries per region sampled. See Table S6 for country data.

Information from the 2018 UN HDI (a combination of life expectancy, school enrollment, literacy, and income, with the lowest human development = 0 and the highest = 1.0) indicates that while the average HDI has increased over the past decades in all primate regions, Madagascar and countries in mainland Africa and South Asia have remained consistently low and below the world’s average (Fig. 6). The HDI for the Neotropics and Southeast Asia has tended to increase, but this has been driven by a small set of countries including Japan, Brunei, Singapore, Taiwan, Argentina, and Costa Rica (Fig. 7). These are among the least primate-rich countries per region. In contrast, the average HDI values for the 25 top developed nations in the world have consistently increased over the same period and are well above the average of all primate range regions (Fig. 7).

Citizens of the nations that rank higher on this index have a higher level of education, a longer lifespan, and a higher gross national income per capita than citizens of nations with a lower HDI (UNDP, 2020). Low levels of development are commonly associated with political instability, extreme income inequality, and limited environmental protection (Alsamawi et al., 2014; UNDP, 2019). Thus, it is clear that despite the enormous biological wealth and natural resources of primate range countries, poverty, deep-rooted income inequality, illiteracy, low levels of education, political instability, and over-exploitation of their natural resources by the consumer nations of the world make the task of preserving primates and their habitats extremely difficult (Alsamawi et al., 2014; UNDP, 2019).

**Figure 8:**
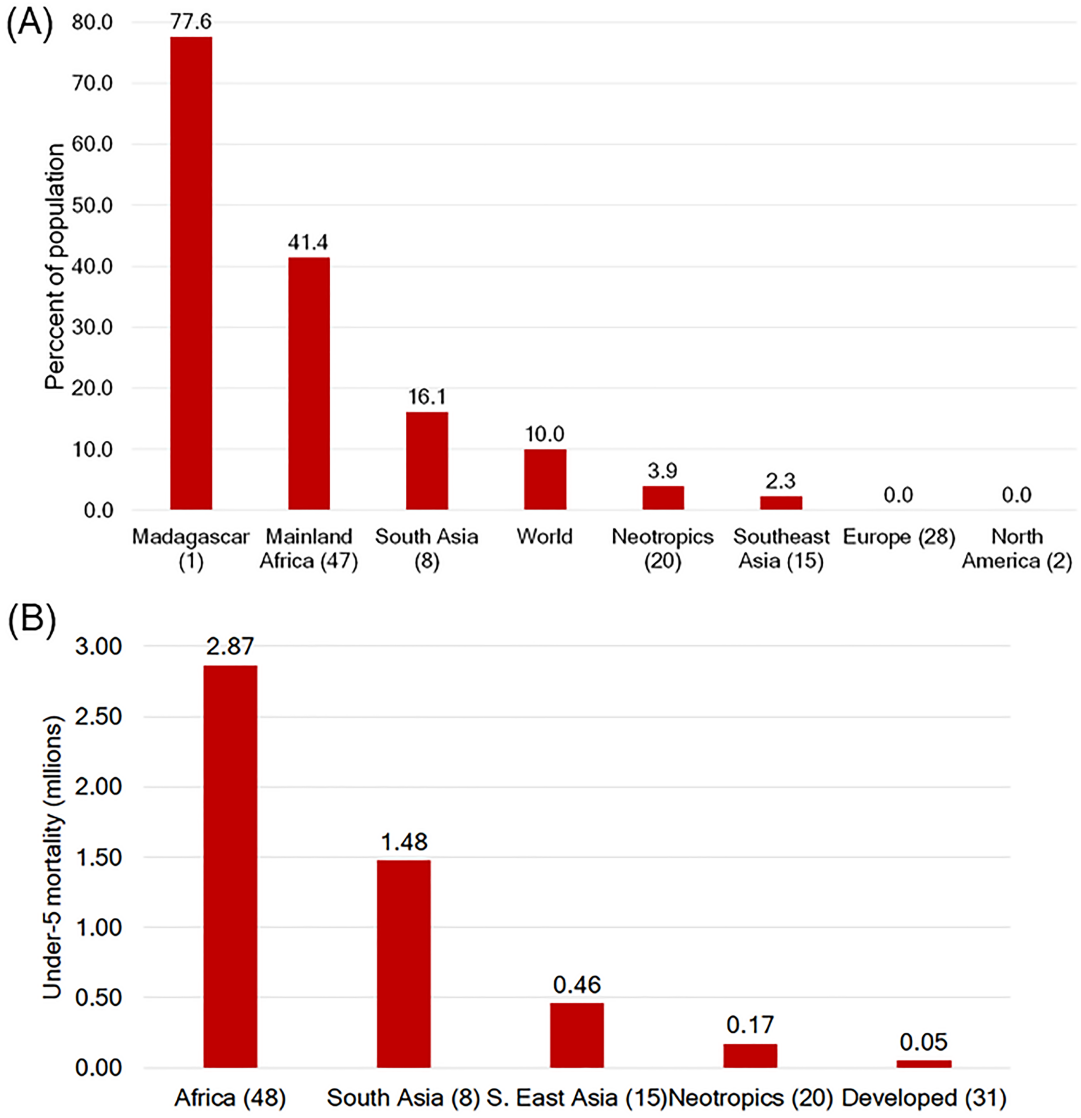
Poverty and under-five mortality in primate range regions. (A) Poverty levels in primate range regions as indicated by the percent of the population living on less than US $1.90 a day. (B) 2018 Under-five mortality in primate range regions. In (B) Madagascar is included with the values from mainland Africa. Source; the World Bank https://data.worldbank.org/indicator/SI.POV.DDAY; https://data.worldbank.org/indicator/SH.DTH.MORT; UNIGME, 2018. Numbers after/under the name of each region refer to the number of countries. See Table S7 for data.

The challenges of devoting sufficient resources to environmental and biodiversity conservation in the majority of primate range countries is further underscored by the percentage of the population living on less than US $1.90 a day, an internationally recognized indicator of extreme poverty (World Bank, 2020b). The World Bank indicates that in 2015 Madagascar had the largest percentage (77%) of the population living on less than US $1.90 a day, followed by mainland Africa (41%) and in South Asia (14%) (Fig. 8A). Individuals in the Neotropics (3.9%) and in Southeast Asia (2.3%) have fared better, and are below the world’s average (10%). In Europe (*n* = 28), the US and Canada the proportion of the population living on less than US$1.90 a day was essentially zero (World Bank, 2020b; Fig. 8A).

Moreover, low human development and high poverty levels in primate range regions are related to high levels of mortality for children under five years of age. In 2018, under-five mortality in mainland Africa was 2.86 million. In South Asia, it totaled 1.47 million children (Fig. 8B). In the Neotropics and in Southeast Asia under-five mortality was significantly lower, 0.17 million and 0.46 million, respectively. In contrast, in developed nations under-five mortality was 0.015 million (Fig. 8B; Table S7) (World Bank, 2020c; UNIGME, 2018). High levels of poverty and its consequences for under-five mortality underscore the challenges faced in prioritizing primate conservation in primate range regions. Poor governance promotes poverty, civil unrest, food insecurity unsustainable use of natural resources, hunting, the illegal wildlife trade, and the necessity to colonize intact forest areas, including legally designated protected areas, thus expanding pressure on primate habitats and populations (Adams & Hutton, 2007; Kates & Dasgupta, 2007).

#### Food security

Food security in primate range countries is a critical factor affecting primate conservation. High levels of population growth, poverty, inequality, and corruption, accompanied by extensive loss of natural capital, have a direct impact on food security. The World Food Summit of 1996 defined food security as the situation in which people have physical, social and economic access to sufficient and nutritive food that meets their dietary requirements for a healthy and active life (http://www.fao.org/WFS/). Using this internationally accepted standard, the GFSI of The Economist Intelligence Unit Limited (GFSI, 2019; https://foodsecurityindex.eiu.com/Index) has integrated the core issues of affordability, availability, quality and safety into a quantitative and qualitative model that evaluates these drivers of food security in developing and developed countries. The index also considers a country’s exposure to the impacts of a changing climate; its susceptibility to natural resource risks; and how the country is adapting to these risks. The GFSI is based on data from 113 countries and ranges from zero (lowest food security) to 100 (highest food security) (https://foodsecurityindex.eiu.com/Index).

**Figure 9:**
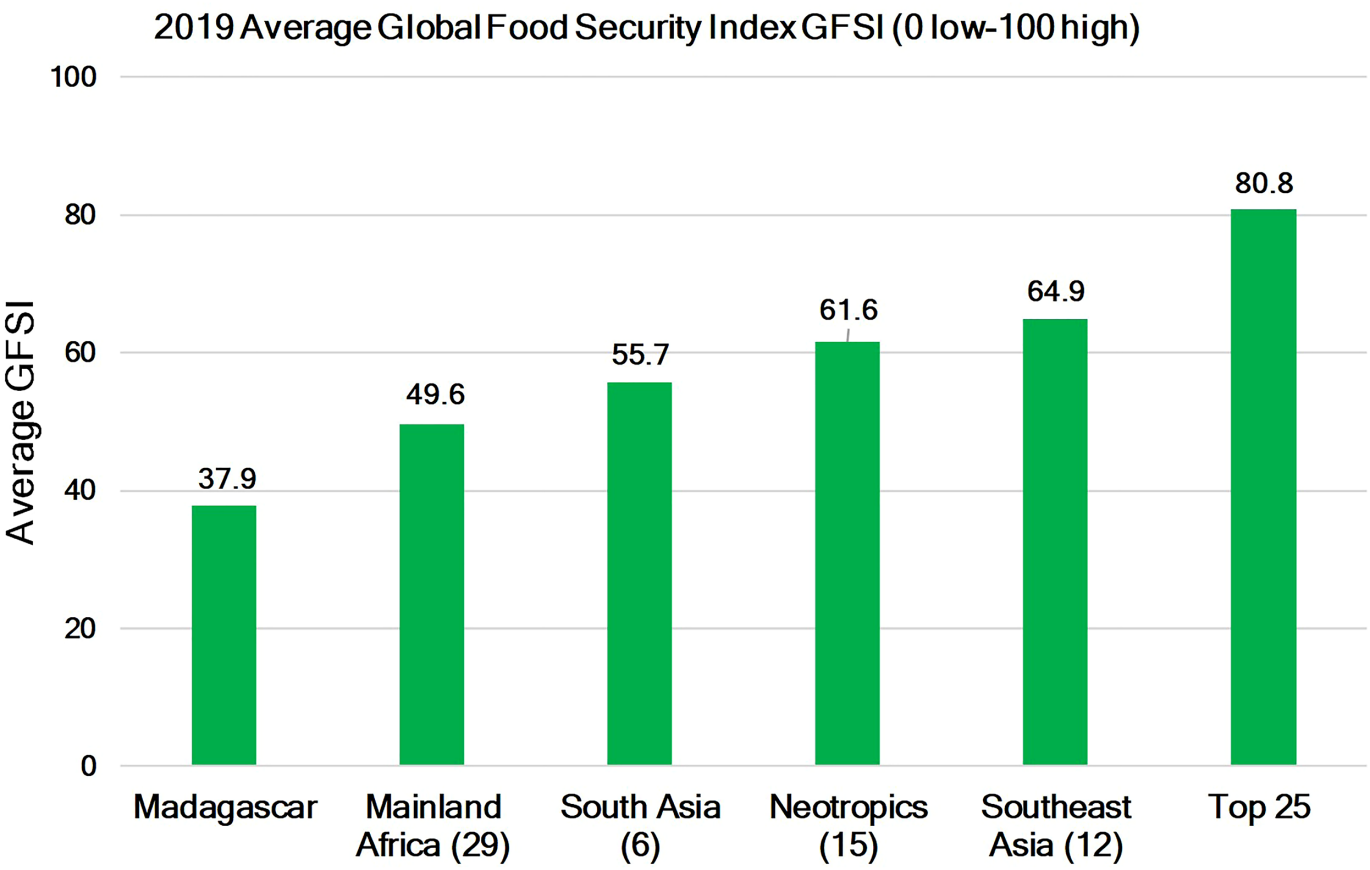
Food security in primate range regions. Global Food Security Index (GFSI) in 2019 for primate range regions (zero is lowest food security to 100 which is highest food security). Also shown is the value of the GFSI for the top 25 most developed nations in the world for which GFSI data are available. See Table S8 for a list of countries and their FSI. Numbers in parentheses represent the number of countries per region for which data are available. See Table S5 for country data.

The GFSI for primate range countries for which the index is available indicates that lowest values are found for Madagascar and mainland Africa, followed by South Asia, the Neotropics, and Southeast Asia. A few Southeast Asian nations such as Japan, Singapore and Malaysia have food security indices comparable to those of Western nations. The average value of the index for primate range regions was 53.9, while the GFSI for 25 of the most developed nations in the world was 80.8 (Fig. 9; Table S8). The United Nations expects that in 2020, 47 of 91 primate range countries (52%), the majority of which are in Africa, will encounter acute food insecurity (GNAFC/FSIN, 2020). Given the economic effects of the coronavirus, it is likely that food insecurity in other primate regions also will increase (Torero-Cullen, 2020). Human populations in primate range countries lag far behind developed nations in GDPPC, in human development, and in food security despite the fact they produce billions of metric tonnes of food per year. This is a direct result of land use practices that are controlled by a small set of multinational corporations and a system of industrial agricultural production for global export (including beef) to satisfy overconsumption by developed and developing nations rather than for domestic consumption (Estrada, Garber & Chaudhary, 2019; Chen, Chaudhary & Mathys, 2020).

Here it is important to note that poverty, corruption, poor governance, and income inequality in many primate range countries, along with debt that must be repaid in dollars, have led countries to allow large areas of their land converted to agricultural and nonagricultural commodities production (e.g., soy fields, oil palm and rubber plantations and the extraction of minerals, fossil fuels and gems) mainly for global markets (Bradshaw & Brook, 2014; Chen et al., 2020; Dietz & Rosa, 1997; Estrada, Garber & Chaudhary, 2019). Land use dedicated to the production and export of food and nonfood commodities by primate range nations has not increased local food security, human safety, or political stability (FAO, 2019a, 2019b).

Adding to the global challenges of income inequality, access to adequate food and health care, conflict, climate impacts, and unforeseen events like the COVID-19 pandemic are expected to have a greater negative impact on health and food security in primate range nations than in more prosperous countries (GNAFC/FSIN, 2020). COVID-19 is expected to overwhelm civil society, increase food insecurity, and devastate the healthcare systems and livelihoods of millions of citizens who work in the informal agricultural and nonagricultural sectors of the economy across the globe (GNAFC/FSIN, 2020). The COVID-19 pandemic has resulted in the disruption of local and global food chains and this may result in scarcities of food and higher prices (Torero-Cullen, 2020), which in turn may lead to increased hunting of bushmeat and an expansion of wildlife trade in many primate range nations (Lappin et al., 2020).

Clearly, primate-range countries must develop a more balanced set of national priorities to build their internal economies in order to ensure food security for their growing human populations. These same countries must also safeguard their biodiversity by building their economies using sustainable practices, green technologies, reducing water, air and soil pollution, and mitigating the effects of climate change on their citizens and environment (Estrada et al., 2017; Sillman et al., 2019). While poverty, food security and protection of primate habitats are intricately linked, there are other social factors at play that impede human well-being, protecting biodiversity, and prioritizing primate conservation. These are corruption and poor governance.

#### Corruption

Corruption is a major threat to humans, biodiversity, and the environment because it destabilizes democratic institutions, causes governmental and societal uncertainty, and destroys public trust. Corruption rewards criminal activity, weakens economic development, promotes inequality, and hinders country-wide prosperity (UNDOC, 2020). Corruption adversely affect human communities and leads to policies and practices that foster habitat degradation and loss of biodiversity. It also contributes to poverty and to social and political volatility. The more a country’s political system is affected by corruption, the poorer the country’s environmental performance (Murshed & Mredula, 2018; Transparency International, 2020).

**Figure 10:**
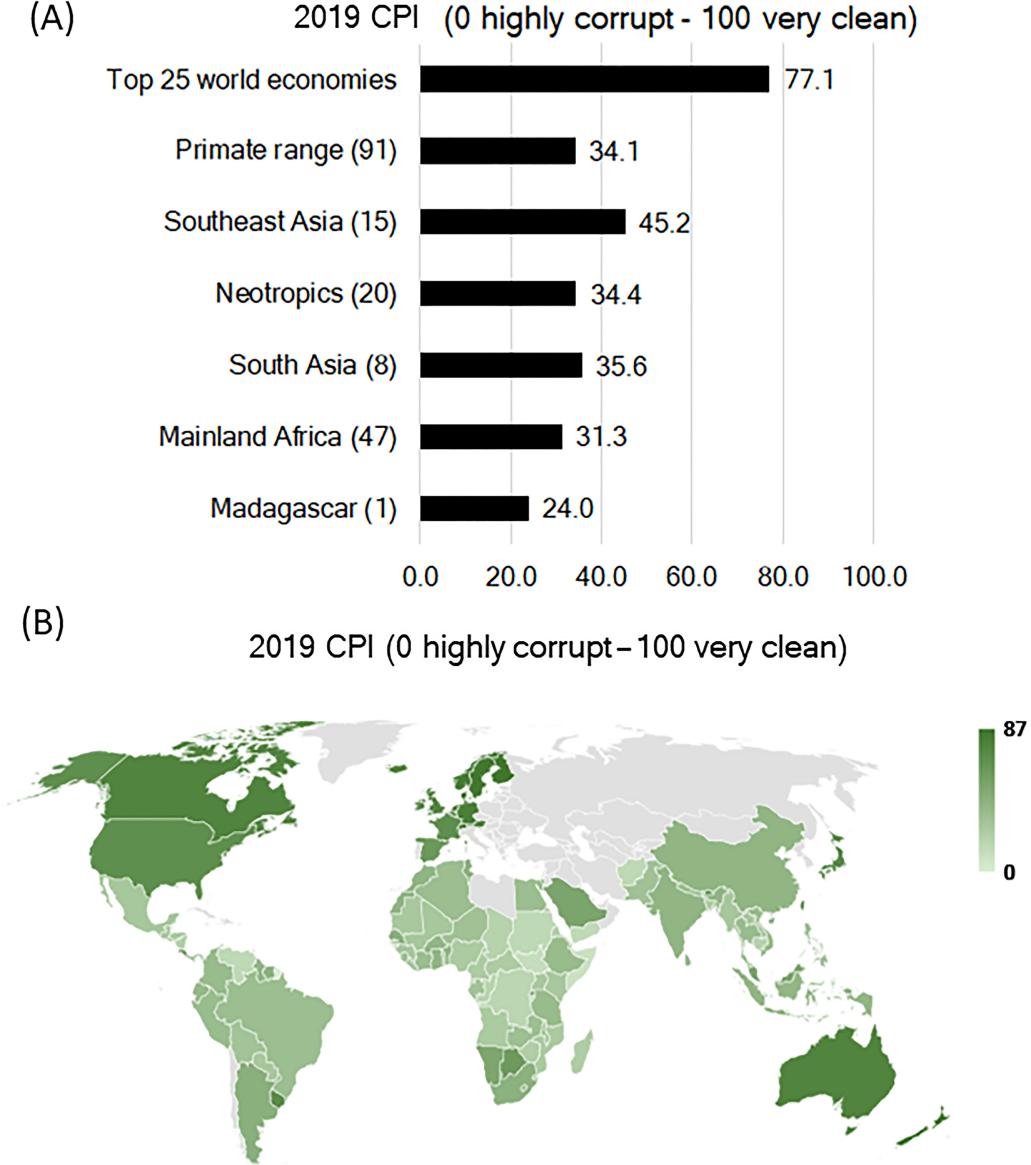
Corruption Perception Index (CPI) in primate range regions. (A) Average Corruption Perception Index (CPI: 0 is highly corrupt to 100 which is very clean) for primate range regions and for the top 25 developed nations for which CPI values are available. The numbers in parenthesis after the name of each region indicate the number of countries sampled. Source: Transparency International www.transparency.org/cpi Consulted March 2020. (B) Map showing the distribution of the 2019 CPI for each primate range country (*n* = 91) and for the 25 most developed nations in the world. See Table S9 for the complete list. Source of data to build map: Transparency International www.transparency.org/cpi. Consulted March 2020. No data are shown for non-primate range countries (shown in gray). See Table S9 for country data.

The Corruption Perceptions Index (CPI), which has been released annually by Transparency International since 1995, ranks countries by their perceived levels of public sector corruption, as validated by expert evaluations and attitude surveys. The CPI defines corruption as “the misuse of public power for private benefit” (Table S9). The 2019 Perception Corruption Index (PCI) of Transparency International (https://www.transparency.org; 0 = most corrupt, 100 = least corrupt) for primate range countries, for which PCI scores are available, and for the top 25 most developed countries indicates that high levels of corruption are widespread in primate range nations compared to the top 25 economies (Fig. 10A). The distribution of the 2019 PCI scores across primate range countries and in the 25 developed nations is shown in Fig. 10B. In 2019, the least corrupt country in the Neotropics was Costa Rica (CPI: 56) followed by Argentina (CPI: 45) and Suriname (CPI: 44). The most corrupt country was Venezuela (CPI: 16) (Fig. 10B; Table S9). In mainland Africa, Botswana, Rwanda and Namibia were the least corrupt (CPI: >50) and the most corrupt were Somalia (CPI: 9), South Sudan (CPI: 12), Equatorial Guinea (CPI: 16), Sudan (CPI: 16), Congo DR (CPI: 18), Guinea-Bissau (CPI: 18), Burundi (CPI: 19) and Republic of Congo (CPI: 19). For the remainder of mainland African countries, the CPI ranged from 20-45. Primates species-rich Madagascar had a CPI score of 24, indicating very high levels of corruption (Fig. 10B; Tables S9). In South Asia the CPI ranged from 15 in Yemen to 68 in Bhutan. In Southeast Asia the CPI ranged from 29 Myanmar/Lao to 85 in Singapore (Fig. 10B; Table S9).

Corruption is a major contributor to primate population decline because it results in incentives to misrepresent the negative consequences of environmental degradation and/or not comply with environmental laws. This has resulted in illegal deforestation and land speculation fostering poverty and criminal activities by individuals and corporations. In many cases these criminal enterprises are associated with the mining of precious metals and gems, which pollutes streams, lakes, rivers, and soil and promotes hunting, logging in protected areas and outside of government concessions, poaching, and the illegal primate pet trade (Human Rights Watch, 2019; Laurance, 2004; Estrada et al., 2018; Estrada, Garber & Chaudhary, 2019). Corruption hinders the conservation efforts of NGOs, governments, and local communities and undermines the capacity of guards and law enforcement to combat drivers of primate habitat loss and local species extirpation (Ivory, 2017; Packer & Polasky, 2018).

In many cases, laws are disregarded through bribery and extortion. In Madagascar, the illegal harvest and export of rosewood in protected areas has been enabled by political ineffectiveness and corruption (Freudenberger, 2010; Gore, Ratsimbazafy & Lute, 2013; Randriamalala & Liu, 2010; Schwitzer et al., 2014). Bushmeat and the trade for body parts are important drivers in the population decline of great ape species—bonobos, chimpanzees, and gorillas in Africa and orangutans in Indonesia (IUCN Red List, 2019). It was estimated that the domestic trade in primates for pets and bushmeat in Peru is likely to number in the hundreds of thousands per year, with larger-bodied primates being the main targets (Shanee, Mendoza & Shanee, 2017). Trading orangutans in Indonesia is a felony, but 440 confiscations in the last 25 years have led to only seven convictions with light sentences (Nijman, 2017). In short, corruption at various levels of society render ineffective the laws that protect wildlife and feed a vicious cycle across many levels of society. High levels of corruption seem to have an important impact on the quality of governance in primate range regions and make social reforms needed to enhance the health, income, and well-being of average citizens, impossible.

#### Governance indicators and implications for primate conservation

**Figure 11:**
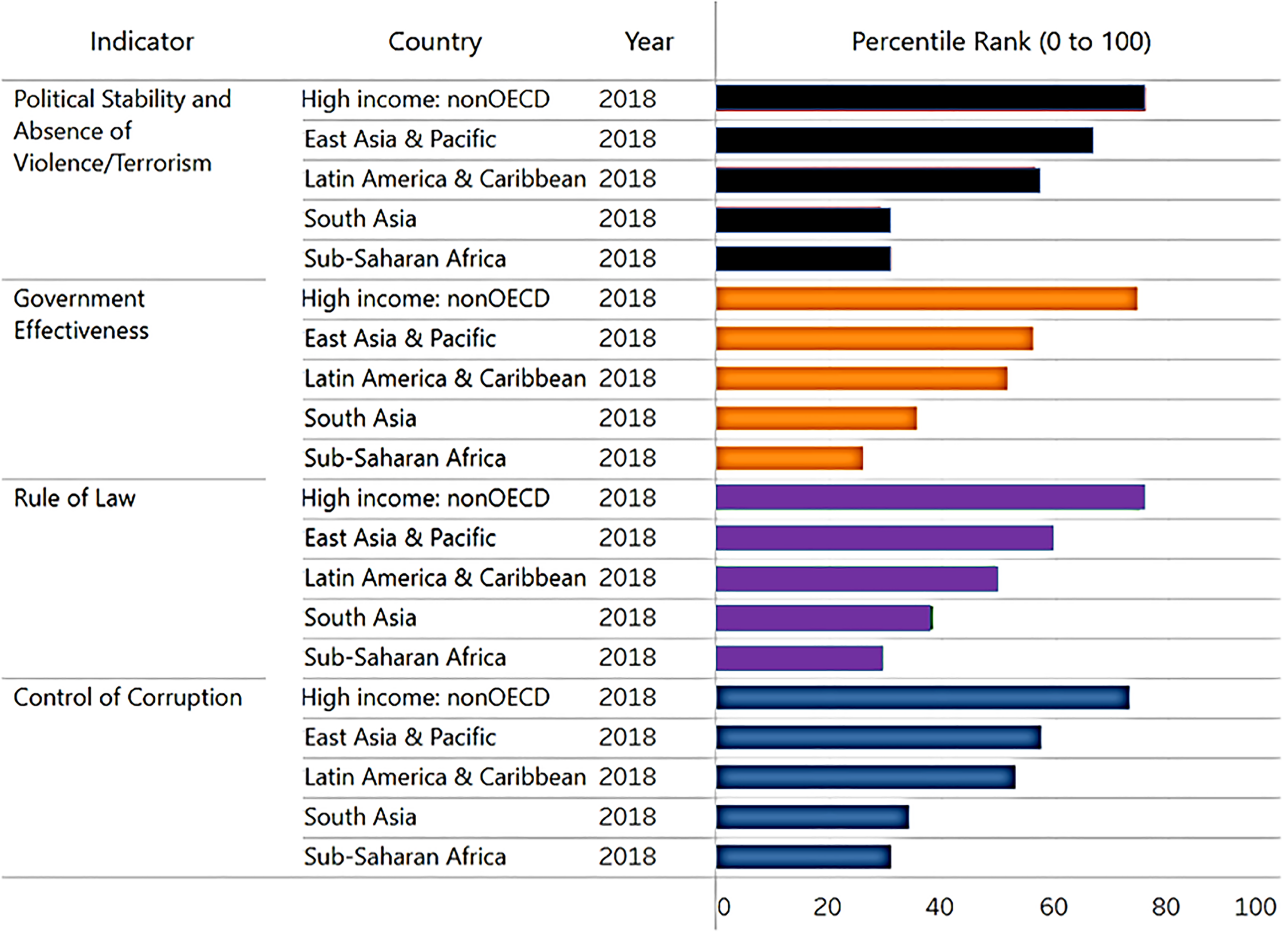
Governance quality in primate range regions. World Bank indicators of governance quality in 2018. This chart shows the percentile rank of each government indicator for each primate range region. East Asia and Pacific refers to Southeast Asia. Madagascar is included under Sub-Saharan Africa. Higher values indicate better governance ratings. Percentile ranks have been adjusted to account for changes over time in the set of countries covered by these governance indicators. Shown for comparison is the percentile rank for high-income non-OECD countries (*n* = 35; Organization for Economic Co-operation and Development). Source of information: http://info.worldbank.org/governance/wgi/Home/Reports. Consulted March 2020.

Good governance entails justly implementing the practices and laws that maintain the institutions and constitution of the country. This involves the process by which governments are chosen, scrutinized and replaced; the capability of the government to successfully create and apply sound policies; and the respect of people and the state for the institutions that govern social and economic interactions (World Bank, 2020d; European Commission, 2019). An examination of four key World Bank indicators of governance quality in 2018 (political stability and absence of violence/terrorism, government effectiveness, rule of law and control of corruption) found that all countries in primate range regions ranked significantly below the average value for 35 high-income countries. Sub-Saharan Africa and Madagascar ranked lowest among primate regions (Fig. 11). A global study showed that high governance scores frequently relate to lower rates of deforestation (Fischera, Giessenb & Günterc, 2020). Rule of law and control of corruption are critical factors in successfully establishing effective conservation programs. Countries will not be successful in implementing policies of conservation and environmental protection if government effectiveness and political stability are low. Irrespective of environmental laws and public sentiment, weak governance fosters the inability of environmental institutions to protect animal and plant biodiversity, safeguard ecosystems health, and promote a green economy.

#### Civil conflict and primate conservation

Tropical forests are one of the earth’s last frontiers. They are rich in natural resources, and they enable residents to maintain their traditional beliefs, economies, and cultures in balance with the environment. Many indigenous cultures view tropical forests as sacred places and view their role as stewards of the environment (Fuentes, 2012). Tropical forests are places of dynamic social, ecological, political, and economic change, which in some cases has led to armed conflict over forest resources and land (McNeely, 2003, 2008).

**Figure 12:**
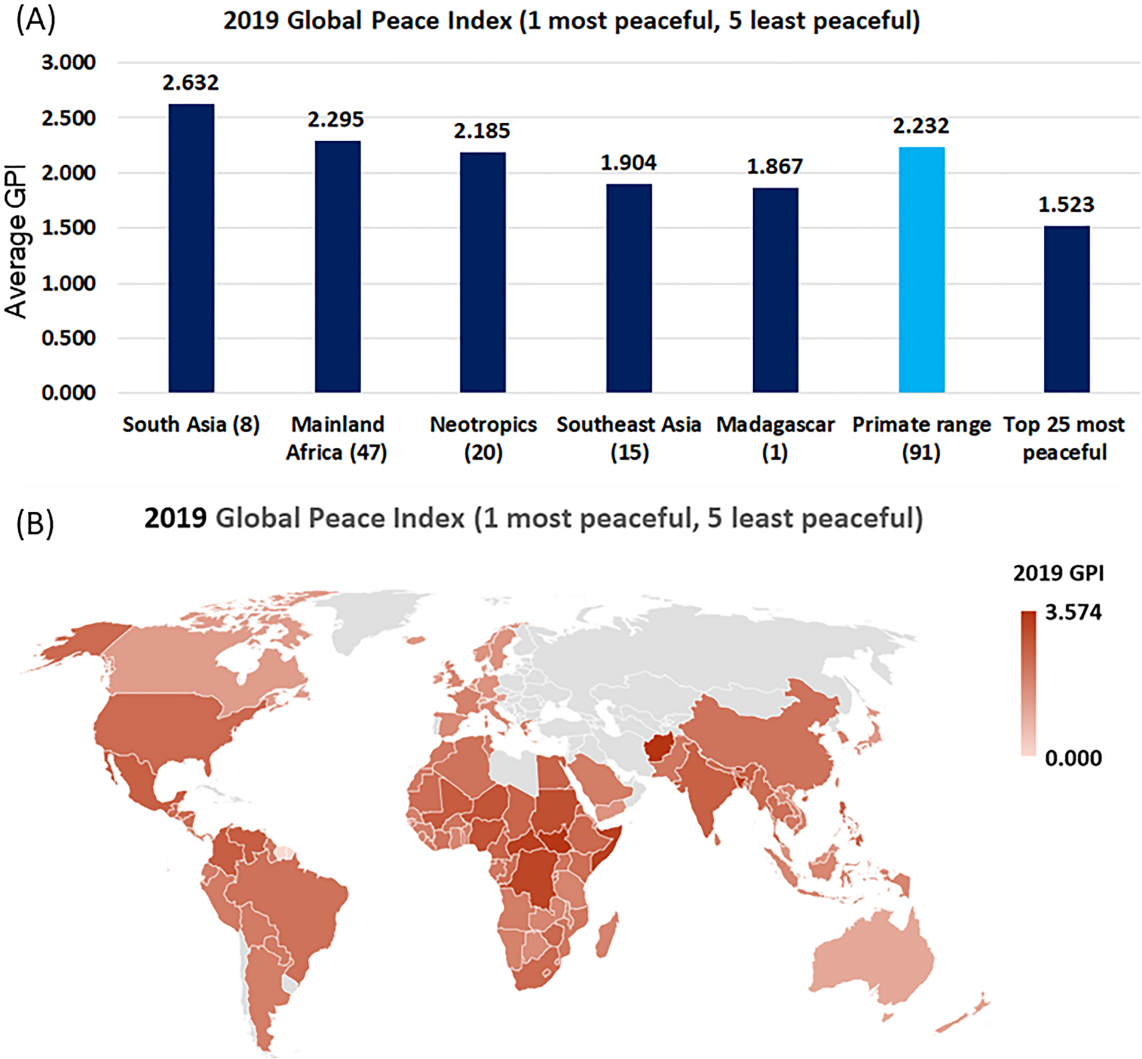
Global Peace Index (GPI) in primate range regions. (A) 2019 Global Peace Index (GPI: 1 is most peaceful and 5 least peaceful) of the Institute of Economics and Peace (https://economicsandpeace.org/). Data are only presented for countries harboring primates and for the top 25 most peaceful countries. Countries in gray, which include northern continental Asia and Libya in north Africa, were not considered because they do not harbor primates. Values in parentheses indicate the number of countries per region included in the analysis. (B) Map showing the distribution of 2019 GPI values for each primate range country and for each of the top 25 most peaceful countries. These 25 countries had the highest 2019 GDPPC. See Tables S5 and S10 for country listings.

Here we examine the 2019 GPI of the Institute of Economics and Peace (https://economicsandpeace.org/). The GPI covers 99.7 per cent of the world’s population and uses 23 qualitative and quantitative indicators to measure the state of peace using three thematic domains, Ongoing Domestic and International Conflict (ODIC), Societal Safety and Security (SSS) and Militarization (IEPGPI, 2019). The 2019 GPI (one most peaceful, five least peaceful) for 91 countries harboring primates had, on average, higher values (GPI: 2.232) than did the top high-income 25 nations in the world (GPI: 1.523) (Fig. 12; Table S10), suggesting that primate countries more commonly face sustained civil conflict. Countries in South Asia and mainland Africa maintain the greatest conflict, followed by countries in the Neotropics. Among primate range nations, countries of Southeast Asia (GPI: 1.904) and Madagascar had the lowest values of the GPI (GPI: 1.867) (Fig. 12).

Civil conflict negatively affects primate population persistence due to random bombing, the use of toxic chemicals and defoliants, increased availability of firearms, and the upsurge in bushmeat hunting both by soldiers and displaced people as local supply chains breakdown (Douglas & Alie, 2014; Loucks et al., 2009). For example, the poaching of bonobos (*Pan paniscus*) and gorillas (*Gorilla gorilla* and *Gorilla beringei*) increased considerably in DRC and Rwanda as a result of ongoing civil wars (Douglas & Alie, 2014). In Cambodia, armed conflicts have severely affected populations of the black-shanked douc (*Pygathrix nigripes*) (Loucks et al., 2009). Heavily armed militias in DRC are currently fighting for ethnic and political control and, jointly with illegal miners, prospect for “conflict minerals” (e.g., coltan, tin, tantalum, tungsten and gold) and diamonds, and hunt primates for bushmeat (Gavin, 2017; Nellemann, Redmond & Refisch, 2010). Likewise, past border conflicts in Southeast Asia, including the war in Vietnam which lasted some 20 years, caused significant damage to the forest and wiped out entire wildlife populations (McNeely, 2003). At present 86% (19/22) of primate species in Vietnam are considered threatened (Table S1).

Civil conflict also alters traditional land use patterns and can lead to increased unregulated forest conversion. In northern Sumatra, between 1990 and 2010 human conflicts combined with forest fires and illegal and legal logging caused major reductions in forest cover (>30%) (Margono et al., 2012). Disputes over land rights, actions by corporation, and governmental policies also have led to forest burning and land-clearing in several primate range regions in Southeast Asia, Africa and the Neotropics endangering many primates (Lanjouw, 2014; Meijaard & Nijman, 2000; Supriatna et al., 2017; Human Rights Watch, 2019).

Clearly, civil unrest, inter-country wars, terrorism, and continued militarization contribute to the dislocation of the large numbers of innocent civilians resulting in refugee crises, increased poverty, insecurity, the spread of disease, environmental damage, and reduced food security. Under these conditions, primate conservation is not a priority and the lack of security and personal safety of citizens in these countries are amplified by prevailing corruption and low-quality governance (Figs. 10 and 11).

### Socioeconomic pathways and primate conservation

**Figure 13:**
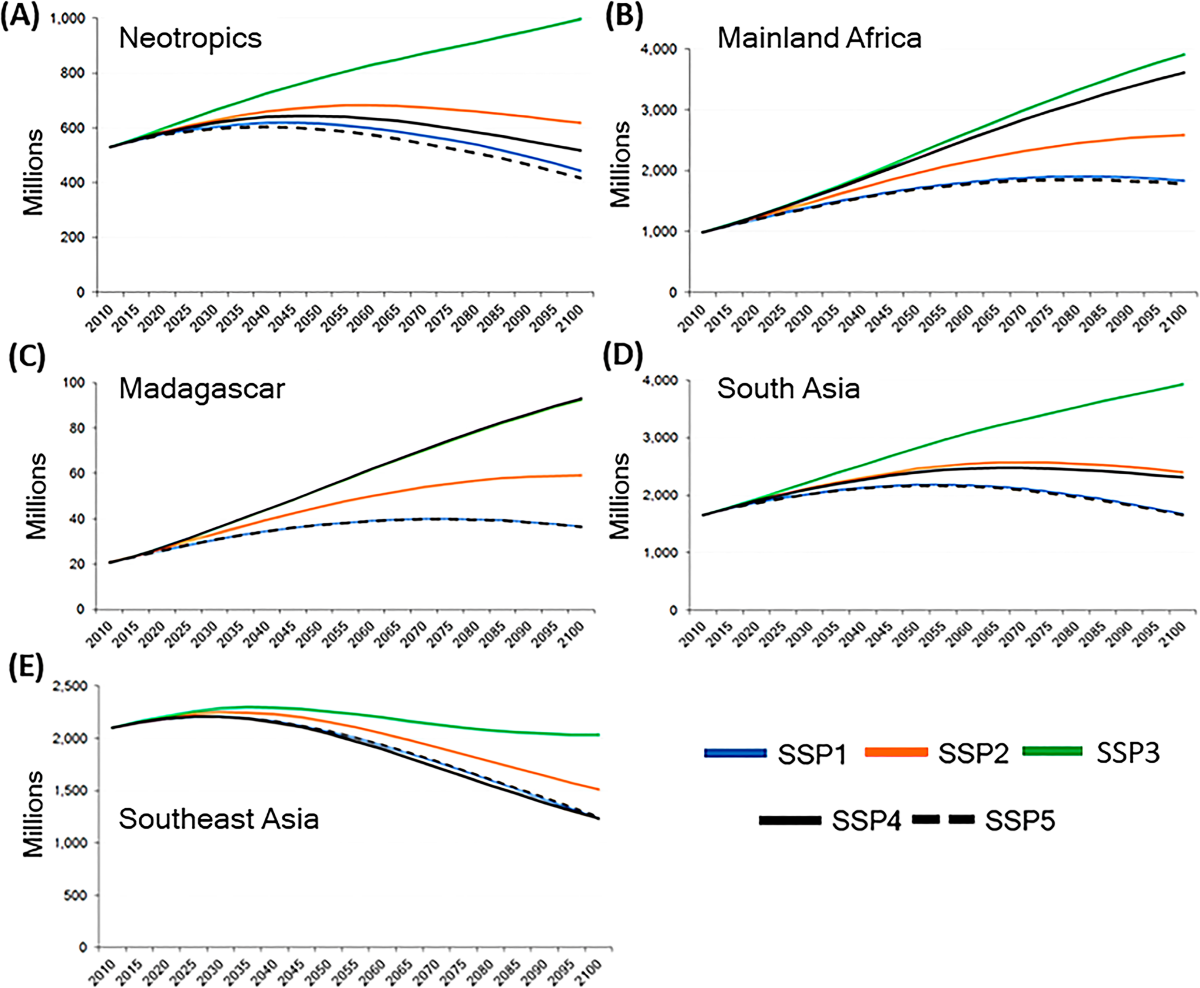
Human population in primate range regions and Shared Socioeconomic Pathways scenarios (SSPs),. Human population projections in primate range regions under five Shared Socioeconomic Pathway (SSPs) scenarios from 2010 to 2100. Source of data: IIASA SSPd database. (https://tntcat.iiasa.ac.at/SspDb/dsd?Action=htmlpage&page=about). These pathways include: (A) a world of sustainability-focused growth and equality (SSP1); (B) a “middle of the road” world where trends broadly follow their historical patterns (SSP2); (C) a fragmented world of “resurgent nationalism” (SSP3); (D) a world of ever-increasing inequality (SSP4); and (E) a world of rapid and unconstrained growth in economic output and energy use (SSP5). See Table S11 (Excel file) for data. *Y* axis refers to human population in millions. Numbers of the *X* axis indicate years.

Decadal interval data (2010–2100) compiled and modeled from all primate countries under five different shared socioeconomic pathways (SSP-1 to SSP-5) (Samir & Lutz, 2017) showed that the lowest human population increase is forecasted under conditions of rapid and unconstrained growth in economic output and energy (SSP-5) that would significantly harm the environment as well as under conditions of sustainability-focused growth and economic equality (SSP-1). The greatest increase in human population growth in the five primate range regions is projected under the SSP-3 scenario, in which globalization is fragmented and countries around the world see a “resurgence of nationalism” (Fig. 13; Table S11) (Samir & Lutz, 2017).

The SSP-1 (sustainability—taking the green road) scenario represents a world that together respects and protects the environment and is shifting towards a green and sustainable path. Under this scenario, tropical deforestation is reduced due to strict regulations, crop yields and international trade increase, people reduce their consumption of meat, adopt healthier diets, and obtain the benefits of a cleaner environment (Van Vuuren et al., 2017). Past studies have gathered both quantitative and qualitative evidence on several local and national interventions that can generate positive outcomes for primates and biodiversity in general. For example, shifting to diets (sustainable consumption) with fewer animal-based products and thus a reduced environmental footprint can relieve some of the existing pressure on species’ habitat, resulting in lower species extinction risk (see Willett et al., 2019; Machovina, Feeley & Ripple, 2015; Chaudhary & Krishna, 2019). Similarly, adopting sustainable tropical forest management practices such as reduced impact logging, can reduce species losses and environmental degradation (Chaudhary et al., 2016). In the agriculture sector, improved technology, reduced food loss and sustainable intensification farming practices leading to higher yields can result in the release of more land for biodiversity conservation (Krause & Ness, 2017; Willett et al., 2019). Assuming that continued human population expansion results in a significant increase in the demand for food, in tropical regions characterized by water scarcity, the cultivation of bioengineered drought-tolerant crops can result in increased production without expanding farming into forested areas (Rosa et al., 2020). No net loss (NNL) biodiversity policies, which are design to reconcile the increased need for infrastructure development with biodiversity conservation are also being formulated (Zu Ermgassen et al., 2019). Finally, at the local level, there exist numerous small-scale, but highly successful, interventions that have shown to mitigate the threats to biodiversity (reviewed in Sutherland et al. (2019)).

Under the SSP-5 (fossil fueled development—taking the highway) scenario, there is rapid technological progress, increase in crop yields and international trade and diets are unhealthy with high food waste. Tropical deforestation continues, but at more modest yearly rates in response to recognition of the need for regulation, however the resulting habitat reduction and fragmentation leads to significant declines in biodiversity, including many primate species (Kriegler et al., 2017). Although the rate of human population increase is similar in both the SSP-1 and SSP-5 scenarios, the SSP-1 scenario forebodes better outcomes for environmental justice, human health and global primate conservation (Table S11).

We note that by 2050, there will be *ca* 700 million more people in primate range countries under the SSP-2 (business-as-usual, middle of the road scenario where development happens along historical patterns) scenario compared with SSP-1 scenario (Table S11). Under the worst case SSP-3 scenario (regional rivalry—a rocky road), the increase will be even more severe and there will be *ca* 1.6 billion additional people in primate hosting countries compared to SSP-1 scenario (see Supplemental Excel). The SSP-3 scenario is characterized by trade barriers, nationalism, limited technology transfer leading to stagnant crop yields, almost no land use change regulations and the prevalence of unhealthy diets high in food waste and meat products (Fujimori et al., 2017). This will certainly be the most disastrous scenarios for primates and humans on Earth.

From these population projections under different SSP scenarios, one can infer that unless sustainability measures associated with the SSP-1 scenario are adopted in the coming decade, the activities of the human population will exert harmful pressures on primates leading to a large number of species extinctions by as early as 2050. However, it also is important to consider that while it is generally assumed that global biodiversity and sustainability policies should be designed to promote economic growth, a recent evaluation has pointed out that increased economic growth may not be required to protect biodiversity and increase human prosperity (IPBES, 2019; Otero et al., 2020).

## Conclusions and Key Challenges Ahead

As we have stressed throughout this manuscript, many historical, socio-economic, political, demographic and cultural factors, in addition to population size, affect patterns of resource consumption, environmental degradation and biodiversity loss. The immediate challenges for primate conservation lie in developing sustainable methods of food production, reducing meat consumption, moving toward a greener lifestyle, limiting the threat of emerging diseases, implementing programs of reforestation, reducing food instability and income inequality, and better governance. That said, the increase in human population size predicted for Africa by the end of the century (estimated population size of 4 billion people), will have an extremely negative impact on primate population persistence, especially for Old World monkeys and apes, and will require very different solutions for environmental protection and human well-being than faced in other parts of the world.

Our review reveals that the well-being, health, and security of the human population in primate range countries is of paramount importance if we are to move forward with effective and long-lasting policies to promote primate conservation. However, high levels of poverty, inequality, food insecurity, and the loss of natural wealth triggered by weak governance, and corruption, along with widespread land-cover changes driven by profiteering and the global market demands of a small number of consumer nations and multinational corporations are the catalysts driving both the primate extinction crisis and the persistence of low human development and poverty (Estrada, Garber & Chaudhary, 2019). According to the UN Department of Economic and Social Affairs, the human cost of climate change disasters will fall devastatingly on low-income and lower-middle-income countries (UNDESA, 2019b). Most of these are primate range nations in Africa, South Asia, and Southeast Asia (Xu et al., 2020).

**Figure 14:**
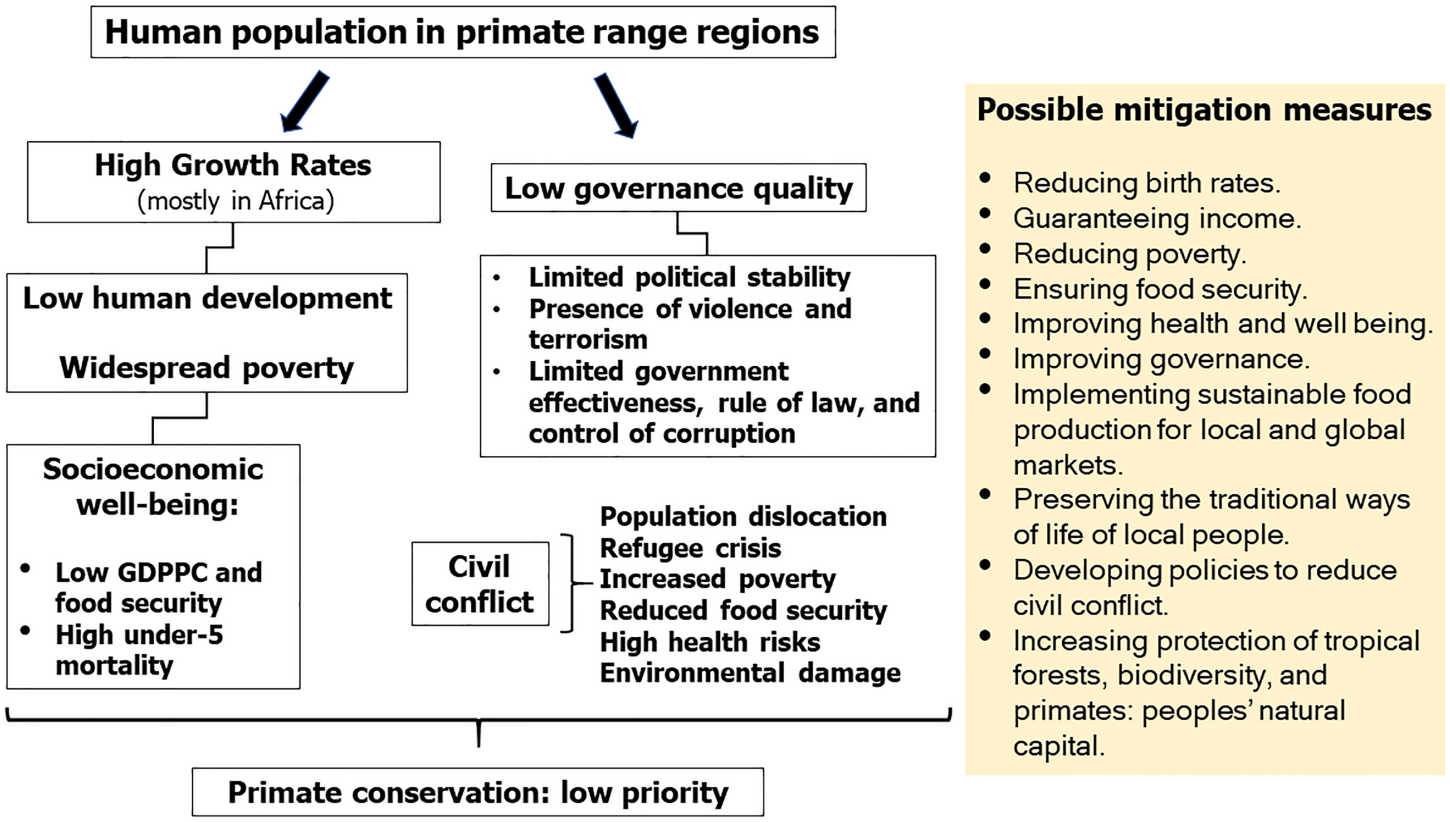
Socioeconomic challenges in primate range regions and primate conservation. Diagram summarizing key socioeconomic challenges facing primate range regions that affect the conservation of their primate fauna. The relative importance of population and governance aspects vary from country to country, but in general these challenges are common to all primate range nations, except Japan, Brunei and Singapore which rank high in Gross Domestic Product per Capita (GDPPC) and in the Human Development Index (HDI) (see Tables S5 and S6).

Solutions to these challenges should involve global approaches to slow human population growth, advance health, lower poverty and improve education, empower women, develop sustainable land-use programs, maintain traditional ways of life of indigenous communities, and adopt green policies of food and natural resource production and consumption, as delineated in the UN Sustainable Development Goals (https://sustainabledevelopment.un.org/#) (Fig. 14). Based on the SSPs-1 model we can accomplish these goals, if global citizens and consumer nations adopt green environmental, economic, and social policies moving forward (Fig. 13).

By 2050, the global population is projected to surge from *ca* 8 to *ca* 10 billion, and estimates are that food production will need to rise from the current 8.4 billion tonnes per year to almost 13.5 billion tonnes (FAO, 2014). The human population in primate range regions is projected to increase from *ca* 6 bn in 2018 to *ca* 8 bn by 2050 adding to the pressures of ensuring food security while at the same time preserving tropical forests to avoid extinctions (Davila & Dyball, 2018; Willett et al., 2019). In the face of globalized market demands, we need global actions to limit the long-term environmental and economic footprint of overconsumption of natural resources from the tropics by citizens in a small number of developed nations as well as an imperative for governments of primate habitat countries to promote human development and well-being (Xu et al., 2020). This will require substantial changes in human behavior in order to provide economic opportunities for the world’s poor by reducing our emphasis on global supply chains and promoting local food production, local manufacture, and local distribution of goods for local markets and consumers. Similarly, we must act to protect tropical forests and the ecosystem services on which agriculture and sustainable use of natural resources depend (FAO, 2014, 2017, 2018, 2019a, 2019b; Willett et al., 2019).

While each region differs from each other in their primate richness and taxonomic diversity, countries in each region have several socioeconomic and sociopolitical traits in common. Our review indicates that all primate range regions are losing tropical forests, and thus natural resources at an alarming rate. Moreover, low human development, low levels of food security and low governance quality are predominant across these regions (Fig. 14).

Although some primate conservation issues, such as climate change, are common to many primate range countries, it is also true that underlying causes of primate population decline vary from country to country, region to region, and differ across primate taxa. Hence, each country will need to identify the set of effective conservation policies and practices required to conserve populations of primate species and their habitats. Moreover, political instability and within country and between country civil unrest are also factors that acting in synergy with other drivers, jeopardize primate conservation, human well-being and the integrity of protected areas (Hammill, 2020).

Despite the collective actions of the world’s countries and international institutions such as the IUCN, UN, World Bank and the UN International Court of Justice, among others, which have led to policies such as the International Convention on Biological diversity (https://www.cbd.int/) and the Paris Climate Agreement (https://unfccc.int/process-and-meetings/the-paris-agreement/the-paris-agreement), biodiversity continues to decline at an accelerated rate (IPBES, 2019). If corrective measures are not soon implemented, we will reach a tipping point and lose our closest living biological relatives along with the complex ecosystem services and benefits they provide to forests and people. We also will lose the social, historical, and cultural relationships that have persisted between human primates and nonhuman primates over millennia (Chandra, 2017; Fuentes, 2012; Voigt et al., 2018).

Moreover, in any discussion of the conservation of natural resources, we need to consider the role played by Indigenous Peoples. According to the UN Department of Economic and Social Affairs and the World Bank there are about 370 million Indigenous Peoples worldwide, living in some 90 countries (most of them in the tropics) (Garnett et al., 2018; Fa et al., 2020; UNDESA, 2020; World Bank, 2020e). Indigenous People inhabit a quarter of the world’s land area and many live in biologically vulnerable environments (e.g., rain forests). Indigenous Peoples hold vital traditional knowledge, and for millennia have sustainably used local natural resources, even in the face of natural disasters (Garnett et al., 2018; Fa et al., 2020; El Bizri et al., 2020; Jarrett, Cummins & Logan-Hines, 2017). And while they represent about five percent of the world’s population and protect about 80 percent of the world’s biodiversity, they account for some 15% of the extreme poor, and their life expectancy is 20 years lower than the life expectancy of nonindigenous peoples worldwide (FAO, 2015; World Bank, 2020d). Acknowledging and protecting the rights of Indigenous Peoples’ to their lands and traditional ways of life are critical if we are to maintain local, regional, and global biodiversity and achieve primate conservation targets (Garnett et al., 2018).

Our review indicates that as human populations are expanding across primate range regions, high levels of poverty, food insecurity and income inequality, among other forces, directly or indirectly, weaken the ability of primate habitat countries to protect tropical forests and biodiversity (Fig. 14). The world community must adopt green technologies and green policies that improve the lives and well-being of poor-and middle-income people and contribute to protect their natural environment. Primates are our closest living biological relatives and as they decline the ecological communities they inhabit also will decline. We are at a historic moment in which urgent local and global action must be taken to reverse the impending extinction of the world’s primates (Estrada et al., 2017). If we can put in place the changes required to save the world’s primates, then we will have put the changes in place to save humans as well.

## Supplemental Information

10.7717/peerj.9816/supp-1 Supplemental Information 1 Tree cover loss (>30% canopy cover) in primate range regions for the period 2001–2018. (A) Neotropics, (B) Africa (Madagascar is included in this graph, but it is distinguished by the yellow bar), (C) Southeast Asia, (D) South Asia. Source of data Global Forest Watch (http://www.globalforestwatch.org; accessed March 2020). Countries ranked by the amount of forest loss.

10.7717/peerj.9816/supp-2 Supplemental Information 2 Primate species present in each country in the regions harboring primates: Mainland Africa, Madagascar, Neotropics, South Asia and Southeast Asia. Listed also is the percent of threatened species and the percent of species with declining populations. Information compiled from the IUCNRedList-2019-3 https://www.iucnredlist.org/. Consulted, March 2020.

10.7717/peerj.9816/supp-3 Supplemental Information 3 Tree cover loss (30% canopy cover) in primate range countries from 2001 to 2018. Source: Global Forest Watch http://www.globalforestwatch.org. Consulted March 2020. Estimates based on remote sensing.

10.7717/peerj.9816/supp-4 Supplemental Information 4 Human population growth between 1960 and 2018 in countries in primate range regions. Growth projections to 2050 and 2100 are also shown. Source of data on population size: https://data.worldbank.org/indicator/; https://population.un.org/wpp/Download/Standard/Population/ Consulted March 2020. UN population projection medium variant 2050 and 2100 from https://ourworldindata.org/grapher/un-population-projection-medium-variant Consulted March 2020.

10.7717/peerj.9816/supp-5 Supplemental Information 5 Rural and urban population growth between 1960 and 2018 in countries in primate range regions. Source of data: World Bank https://data.worldbank.org/indicator/SP.RUR.TOTL.ZS?locations=DZ-AO-BJ-AR-BW. Consulted March 2020.

10.7717/peerj.9816/supp-6 Supplemental Information 6 Socioeconomic indicators of human well-being in primate regions. 2018 Gross Domestic Product per Capita (GDDPC), 2018 Human Development Index, 2019 Global Food Security Index, and 2019 Corruption Perception Index (CPI) for primate range countries. NA = not available. Sources of data for GDPPC, The World Bank https://data.worldbank.org/indicator/NY.GDP.PCAP.CD. Human Development Index, United Nations Development Programme http://hdr.undp.org/en/data, http://hdr.undp.org/en/data. Global Food Security Index, Economist Intelligent Unit https://foodsecurityindex.eiu.com/Index. Corruption Perception Index, Transparency International www.transparency.org/cpi. Each of these sources was consulted in March 2020.

10.7717/peerj.9816/supp-7 Supplemental Information 7 UN Human Development Index in primate range regions. UN Human Development Index (HDI: 0 low, 1.0 highest). United Nations Development Programme. http://hdr.undp.org/en/data; http://worldpopulationreview.com/countries/hdi-by-country/ Consulted March 2020.

10.7717/peerj.9816/supp-8 Supplemental Information 8 Under-five mortality. 2018 Under-five mortality. Source World Bank https://data.worldbank.org/indicator/SH.DTH.MORT Consulted March 2020. Summaries are shown for primate regions, for top 25 developed nations and for the EU, UK, USA and Canada.

10.7717/peerj.9816/supp-9 Supplemental Information 9 Global Food Security Index. Global Food Security Index (FSI: 0 low, 100 high) of The Economist Intelligent Unit Limited. https://foodsecurityindex.eiu.com/Index. Consulted March 2020.

10.7717/peerj.9816/supp-10 Supplemental Information 10 Perception Corruption Index. Perception Corruption Index (PCI: 0 highly corrupt, 100 very clean) of Transparency International www.transparency.org/cpi. Consulted March 2020. The Corruption Perceptions Index is a composite index, a combination of different international surveys and assessments of corruption, collected by a variety of reputable institutions. The index draws on 13 surveys from independent institutions specializing in governance and business climate analysis covering expert assessments and views of businesspeople. None of these surveys were commissioned by Transparency International (https://www.transparency.org/news/pressrelease/explanation_of_how_individual_country_scores_of_the_corruption_perceptions).

10.7717/peerj.9816/supp-11 Supplemental Information 11 Global Peace Index. Global Peace Index (GPI: 1 most peaceful, 5 least peaceful) of the Institute of Economics and Peace. http://economicsandpeace.org/reports/. Global Peace Index 2019 list of countries is from: Measuring Peace in a Complex World, Sydney, June 2019. Available from: http://visionofhumanity.org/reports (Consulted March 2020).

10.7717/peerj.9816/supp-12 Supplemental Information 12 Human population growwth projecctions under five Shared Socioeconomic Pathways scenarios. Future projections of human population growth and GDP|PPP under five Shared Socio-Economic Pathway Scenarios (SSPs) from 2010 to 2100 for each primate region (the Neotropics, mainland Africa, Madagascar, South Asia and Southeast Asia) were obtained from IIASA SSPd database. (https://tntcat.iiasa.ac.at/SspDb/dsd?Action=htmlpage&page=about).

## Additional Information and Declarations

### Data Availability

The following information was supplied regarding data availability: The raw measurements are available in the Supplemental Files.
