# Supplementary material for: Current and future trends in socio-economic, demographic and governance factors affecting global primate conservation
Source: PeerJ. 2020 Aug 21;8:e9816. doi: 10.7717/peerj.9816 (PMC7444509; doi:10.7717/peerj.9816)
Supplement: Supplemental Information 1 — (A) Neotropics, (B) Africa (Madagascar is included in this graph, but it is distinguished by the yellow bar), (C) Southeast Asia, (D) South Asia. Source of data Global Forest Watch (http://www.globalforestwatch.org; accessed March 2020). Countries ranked by the amount of forest loss. [file peerj-08-9816-s001.docx]

**Figure S1.** Tree cover loss (>30% canopy cover) in primate range regions for the period 2001–2018. **(A)** Neotropics, **(B)** Africa (Madagascar is included in this graph, but it is distinguished by the yellow bar), **(C)** Southeast Asia, **(D)** South Asia. Source of data Global Forest Watch (http://www.globalforestwatch.org; accessed March 2020). Countries ranked by the amount of forest loss.

**(A)**

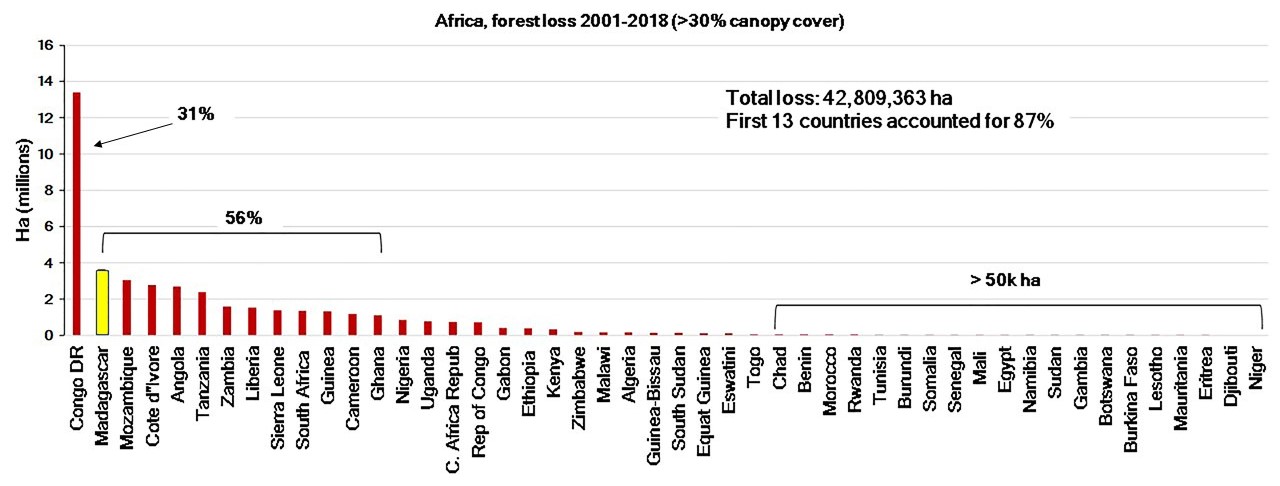
**(B)**

Ha (millions)

**(C)**

Ha (millions)

**(D)**


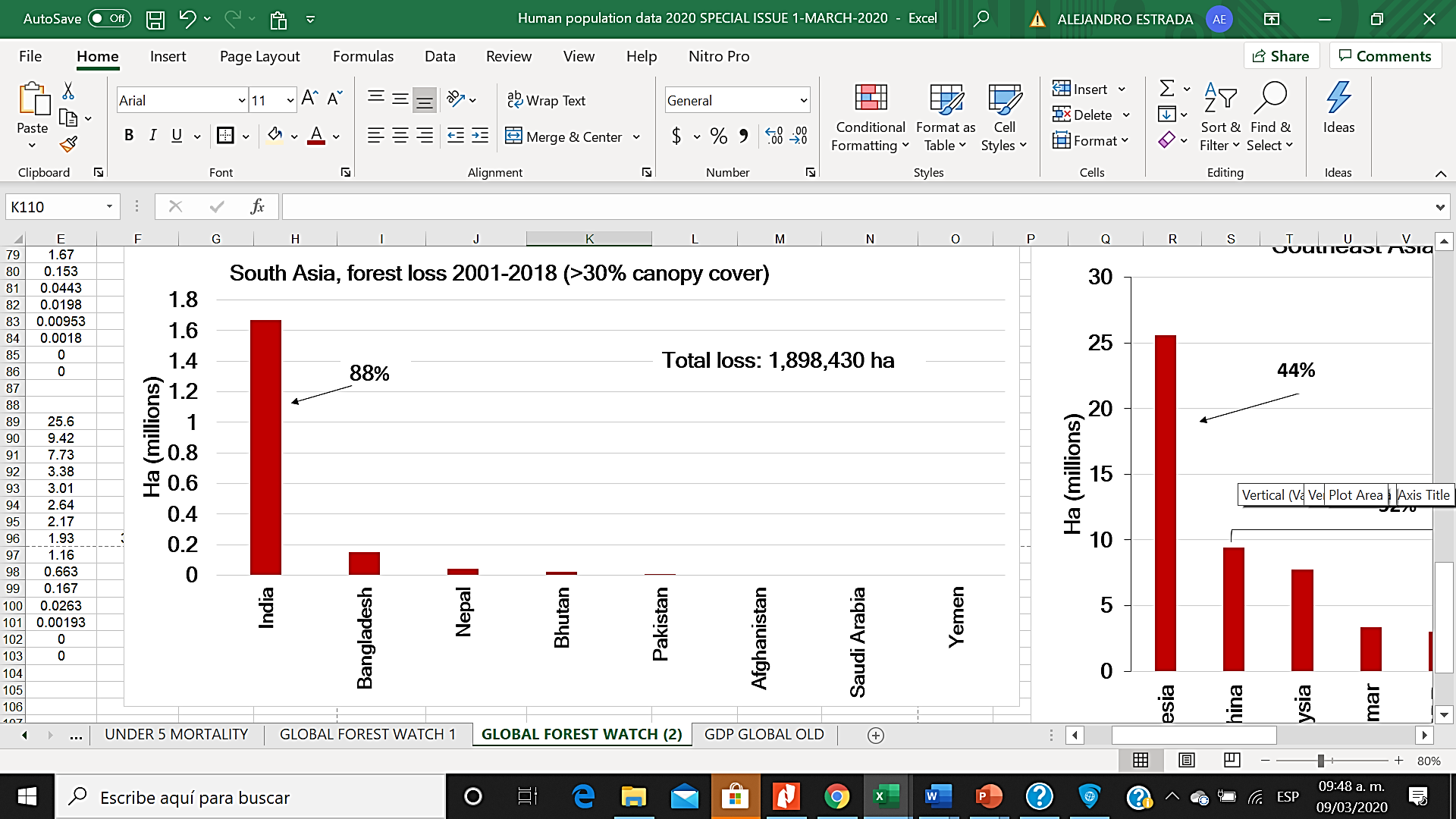


Ha (millions)
