# Supplementary material for: Current and future trends in socio-economic, demographic and governance factors affecting global primate conservation
Source: PeerJ. 2020 Aug 21;8:e9816. doi: 10.7717/peerj.9816 (PMC7444509; doi:10.7717/peerj.9816)
Supplement: Supplemental Information 2 — Listed also is the percent of threatened species and the percent of species with declining populations. Information compiled from the IUCNRedList-2019-3 https://www.iucnredlist.org/. Consulted, March 2020. [file peerj-08-9816-s002.docx]

**Table S1.** Primate species present in each country in the regions harboring primates: Mainland Africa, Madagascar, Neotropics, South Asia and Southeast Asia. Listed also is the percent of threatened species and the percent of species with declining populations. Information compiled from the IUCNRedList-2019-3 https://www.iucnredlist.org/. Consulted, March 2020.

|  |  |  | **%** | **%** |
| --- | --- | --- | --- | --- |
| **Region/Country** | **Species** | **Threatened** | **species threatened** | **pop decreasing** |
| **Mainland Africa** |  |  |  |  |
| Algeria | **1** | **1** | **100** | **100** |
| Angola | **24** | **2** | **8** | **18** |
| Benin | **14** | **5** | **36** | **50** |
| Botswana | **3** | **0** | **0** | **67** |
| Burkina Faso | **9** | **3** | **33** | **56** |
| Burundi | **14** | **4** | **29** | **64** |
| Somalia | **9** | **0** | **0** | **56** |
| Ethiopia | **13** | **1** | **8** | **54** |
| Cameroon | **32** | **10** | **31** | **56** |
| Central African Republic | **22** | **4** | **18** | **55** |
| Chad | **5** | **0** | **0** | **60** |
| Congo | **24** | **8** | **33** | **42** |
| Congo DR | **45** | **14** | **32** | **64** |
| Cote d´Ivoire | **20** | **10** | **50** | **65** |
| Djibouti | 2 | 0 | **0** | **0** |
| Egypt | **1** | **0** | **0** | **0** |
| Equatorial Guinea | **24** | **10** | **42** | **54** |
| Eswatini | **4** | **0** | **0** | **75** |
| Gabon | **21** | **6** | **29** | **62** |
| Gambia | **7** | **1** | **14** | 43 |
| Ghana | **17** | **7** | **41** | **53** |
| Guinea | **17** | **6** | **35** | **47** |
| Guinea-Bissau | **13** | **3** | **23** | **38** |
| Kenya | **21** | **1** | **5** | **57** |
| Lesotho | 1 | 0 | **0** | **100** |
| Liberia | **13** | **5** | **38** | **46** |
| Malawi | **7** | **0** | **0** | **57** |
| Mali | **7** | **1** | **14** | **43** |
| Mauritania | **4** | **0** | **0** | **25** |
| Morocco | **1** | **1** | **100** | **100** |
| Mozambique | **9** | **0** | **0** | **56** |
| Namibia | **3** | **0** | **0** | **33** |
| Niger | **4** | **0** | **0** | **50** |
| Nigeria | **27** | **13** | **48** | **56** |
| Rwanda | **19** | **6** | **32** | **74** |
| Senegal | **13** | **3** | **23** | **50** |
| Sierra Leone | **17** | **5** | **29** | **47** |
| South Africa | **5** | **0** | **0** | **80** |
| South Sudan | **11** | **1** | **9** | **55** |
| Sudan | **6** | **0** | **0** | **33** |
| Tanzania | **27** | **8** | **30** | **67** |
| Togo | **14** | **4** | **29** | **43** |
| Tunisia | **1** | **1** | **100** | **100** |
| Uganda | **20** | **5** | **25** | **70** |
| Zambia | **11** | **0** | **0** | **36** |
| Zimbabwe | **6** | **0** | **0** | **67** |
| Eritrea | **4** | **0** | **0** | **25** |
|  |  |  |  |  |
| **Madagascar** | **100** | **93** | **93** | **97** |
|  |  |  |  |  |
| **Neotropics** |  |  |  |  |
| Belize | **2** | **2** | **100** | **100** |
| Costa Rica | **3** | **2** | **67** | **67** |
| El Salvador | **1** | **1** | **100** | **100** |
| Guatemala | **3** | **2** | **67** | **67** |
| Honduras | **2** | **1** | **50** | **50** |
| Mexico | **3** | **2** | **67** | **67** |
| Nicaragua | **2** | **1** | **50** | **50** |
| Panama | **6** | **3** | **50** | **67** |
| Argentina | **5** | 0 | **0** | **100** |
| Bolivia | **25** | **6** | **24** | **72** |
| Brazil | **120** | 41 | **34** | **68** |
| Colombia | **43** | **22** | **51** | **77** |
| Ecuador | **19** | **7** | **37** | **79** |
| French Guiana | **8** | **1** | **13** | **50** |
| Guyana | **8** | **1** | **13** | **50** |
| Paraguay | **5** | **0** | **0** | **60** |
| Peru | **49** | **13** | **27** | **79** |
| Suriname | **8** | **1** | **13** | **50** |
| Trinidad | 1 | 1 | **100** | **100** |
| Venezuela | **20** | 7 | **35** | **75** |
|  |  |  |  |  |
| **South Asia** |  |  |  |  |
| Afghanistan | **2** | **0** | **0** | **50** |
| Bangladesh | **10** | **6** | **60** | **90** |
| Bhutan | **7** | **2** | **29** | **86** |
| Yemen | **1** | **0** | **0** | **0** |
| India | **23** | **13** | **57** | **91** |
| Nepal | **5** | **1** | **20** | **80** |
| Pakistan | **2** | **0** | **0** | **50** |
| Saudi Arabia | 1 | 0 | **0** | **0** |
|  |  |  |  |  |
| **Southeast Asia** |  |  |  |  |
| Brunei | **10** | **7** | **70** | **100** |
| Cambodia | **11** | **9** | **82** | **100** |
| China | **25** | **19** | **76** | **80** |
| Indonesia | **48** | 40 | **83** | **100** |
| Japan | **1** | **0** | **0** | **0** |
| Lao PDR | **18** | **15** | **83** | **94** |
| Malaysia | **20** | **14** | **70** | **100** |
| Myanmar | **18** | 12 | **67** | **94** |
| Philippines | **3** | **1** | **33** | **100** |
| Singapore | **4** | **2** | **50** | **100** |
| Sri Lanka | **5** | **3** | **60** | **100** |
| Taiwan | **1** | **0** | **0** | **0** |
| Thailand | **18** | 11 | **61** | **94** |
| Timor-Leste | **1** | **0** | **0** | **100** |
| Vietnam | **22** | **19** | **86** | **95** |
