# Supplementary material for: Current and future trends in socio-economic, demographic and governance factors affecting global primate conservation
Source: PeerJ. 2020 Aug 21;8:e9816. doi: 10.7717/peerj.9816 (PMC7444509; doi:10.7717/peerj.9816)
Supplement: Supplemental Information 3 — Source: Global Forest Watch http://www.globalforestwatch.org. Consulted March 2020. Estimates based on remote sensing. [file peerj-08-9816-s003.docx]

**Table S2.** Tree cover loss (30% canopy cover) in primate range countries from 2001 to 2018. Source: Global Forest Watch http://www.globalforestwatch.org Consulted March 2020. Estimates based on remote sensing.

|  | **2001-2018** |  |  | **2001-2018** |
| --- | --- | --- | --- | --- |
| Country | **Ha** |  |  | **Ha** |
| **mainland Africa** |  |  | **Neotropics** |  |
| Algeria | 152,000 |  | Belize | 217,000 |
| Angola | 2,690,000 |  | Costa Rica | 233,000 |
| Benin | 38,200 |  | El Salvador | 74,400 |
| Botswana | 505 |  | Guatemala | 1,390,000 |
| Burkina Faso | 131 |  | Honduras | 1,010,000 |
| Burundi | 25,300 |  | Mexico | 3,670,000 |
| Cameroon | 1,200,000 |  | Nicaragua | 1,400,000 |
| Central Africa Republic | 739,000 |  | Panama | 380,000 |
| Chad | 41,400 |  | Argentina | 5,770,000 |
| Republic of Congo | 712,000 |  | Paraguay | 5,720,000 |
| Congo DR | 13,400,000 |  | Brazil | 53,800,000 |
| Cote d’Ivoire | 2,780,000 |  | Bolivia | 4,830,000 |
| Djibouti | 0 |  | Colombia | 4,070,000 |
| Egypt | 1,710 |  | Peru | 2,880,000 |
| Equatorial Guinea | 108,000 |  | Venezuela | 1,950,000 |
| Eswatini | 94,500 |  | Ecuador | 787,000 |
| Gabon | 407,000 |  | Guyana | 183,000 |
| Gambia | 639 |  | Suriname | 166,000 |
| Ghana | 1,090,000 |  | French Guiana | 68,700 |
| Guinea | 1,320,000 |  | Trinidad and Tobago | 21,300 |
| Guinea-Bissau | 142,000 |  |  |  |
| Kenya | 326,000 |  | **South Asia** |  |
| Lesotho | 90 |  | Afghanistan | 1,800 |
| Liberia | 1,530,000 |  | Bangladesh | 153,000 |
| Malawi | 164,000 |  | Bhutan | 19,800 |
| Mali | 3,300 |  | India | 1,670,000 |
| Mauritania | 33 |  | Nepal | 44,300 |
| Morocco | 36,200 |  | Pakistan | 9,530 |
| Mozambique | 3,050,000 |  | Saudi Arabia | 0 |
| Namibia | 1,220 |  | Yemen | 0 |
| Niger | 0 |  |  |  |
| Nigeria | 858,000 |  | **Southeast Asia** |  |
| Rwanda | 32,000 |  | Brunei | 26,300 |
| Senegal | 3,430 |  | Cambodia | 2,170,000 |
| Sierra Leone | 1,380,000 |  | China | 9,420,000 |
| South Africa | 1,340,000 |  | Indonesia | 25,600,000 |
| South Sudan | 119,000 |  | Japan | 663,000 |
| Sudan | 962 |  | Lao PDR | 3,010,000 |
| Tanzania | 2,370,000 |  | Malaysia | 7,730,000 |
| Togo | 50,750 |  | Myanmar | 3,380,000 |
| Tunisia | 26,400 |  | Philippines | 1,160,000 |
| Uganda | 781,000 |  | Singapore | 1,930 |
| Zambia | 1,580,000 |  | Sri Lanka | 167,000 |
| Zimbabwe | 197,000 |  | Taiwan | --- |
| Ethiopia | 384,000 |  | Thailand | 1,930,000 |
| Eritrea | 3 |  | Timor-Leste | --- |
| Somalia | 3,590 |  | Vietnam | 2,640,000 |
|  |  |  |  |  |
| **Madagascar** | 3,630,000 |  |  |  |
