# Supplementary material for: Current and future trends in socio-economic, demographic and governance factors affecting global primate conservation
Source: PeerJ. 2020 Aug 21;8:e9816. doi: 10.7717/peerj.9816 (PMC7444509; doi:10.7717/peerj.9816)
Supplement: Supplemental Information 4 — Growth projections to 2050 and 2100 are also shown. Source of data on population size: https://data.worldbank.org/indicator/; https://population.un.org/wpp/Download/Standard/Population/ Consulted March 2020. UN population projection medium variant 2050 and 2100 from https://ourworldindata.org/grapher/un-population-projection-medium-variant Consulted March 2020. [file peerj-08-9816-s004.docx]

**Table S3.** Human population growth between 1960 and 2018 in countries in primate range regions. Growth projections to 2050 and 2100 are also shown. Source of data on population size: https://data.worldbank.org/indicator/; https://population.un.org/wpp/Download/Standard/Population/ Consulted March 2020. UN population projection medium variant 2050 and 2100 from https://ourworldindata.org/grapher/un-population-projection-medium-variant Consulted March 2020.

|  | **Human population millions** |  |  |  | **Projections** | **Projections** |
| --- | --- | --- | --- | --- | --- | --- |
| **Country** | **1960** | **1980** | **2000** | **2018** | **2050** | **2100** |
| **mainland Africa** |  |  |  |  |  |  |
| Algeria | 11.0 | 19.0 | 31.0 | 42.0 | 61,928,118 | 70,409,629 |
| Angola | 5.4 | 8.3 | 16.3 | 30.8 | 76,046,053 | 172,860,941 |
| Benin | 2.4 | 3.7 | 6.8 | 11.4 | 23,929,846 | 44,325,028 |
| Botswana | 0.5 | 0.9 | 1.6 | 2.2 | 3,421,404 | 3,773,608 |
| Burkina Faso | 4.8 | 6.8 | 11.6 | 19.7 | 43,207,053 | 81,722,867 |
| Burundi | 2.7 | 4.1 | 6.3 | 11.1 | 25,762,044 | 54,513,584 |
| Somalia | 2.7 | 6.2 | 8.8 | 15.0 | 35,851,792 | 78,971,914 |
| Ethiopia | 22.1 | 35.1 | 66.2 | 109.2 | 190,869,632 | 249,529,919 |
| Cameroon | 5.1 | 8.6 | 15.5 | 25.2 | 49,817,390 | 91,640,537 |
| Central African Rep | 1.5 | 2.1 | 3.6 | 4.4 | 8,850,780 | 14,055,261 |
| Chad | 3.0 | 4.9 | 8.3 | 15.4 | 33,635,795 | 61,690,672 |
| Congo | 1.0 | 1.7 | 3.1 | 5.2 | 11,509,651 | 23,576,569 |
| Congo DR | 15.2 | 26.3 | 47.1 | 84.0 | 197,404,202 | 378,975,244 |
| Cote d’Ivoire | 3.5 | 8.0 | 16.0 | 25.0 | 51,375,178 | 103,563,352 |
| Djibouti | 0.1 | 0.4 | 0.7 | 1.0 | 1,307,615 | 1,263,813 |
| Egypt | 26.6 | 43.3 | 68.8 | 98.4 | 153,433,492 | 198,748,056 |
| Equatorial Guinea | 0.3 | 0.3 | 0.6 | 1.3 | 2,844,744 | 4,740,885 |
| Eswatini | 0.3 | 0.6 | 1.0 | 1.1 | 2,081,036 | 2,457,301 |
| Gabon | 0.5 | 0.6 | 1.2 | 2.1 | 3,516,141 | 4,996,323 |
| Gambia | 0.3 | 0.7 | 1.3 | 2.2 | 4,562,152 | 7,179,802 |
| Ghana | 6.6 | 11.0 | 19.2 | 29.7 | 51,269,943 | 76,754,605 |
| Guinea | 3.9 | 4.8 | 8.2 | 12.4 | 26,852,336 | 48,326,902 |
| Guinea-Bissau | 0.6 | 0.8 | 1.2 | 1.8 | 3,602,736 | 5,900,589 |
| Kenya | 8.1 | 16.4 | 31.8 | 51.3 | 95,467,137 | 142,123,899 |
| Lesotho | 0.8 | 1.3 | 2.0 | 2.1 | 3,203,470 | 3,904,989 |
| Liberia | 1.1 | 1.8 | 2.8 | 4.8 | 9,804,031 | 17,800,502 |
| Malawi | 3.6 | 6.2 | 11.1 | 18.1 | 41,705,002 | 75,710,602 |
| Mali | 5.2 | 7.0 | 10.9 | 19.0 | 44,020,171 | 83,207,100 |
| Mauritania | 0.9 | 0.5 | 2.6 | 4.4 | 8,965,000 | 15,518,953 |
| Morocco | 12.3 | 20.0 | 28.7 | 36.0 | 45,659,886 | 43,840,152 |
| Mozambique | 7.1 | 11.6 | 18.2 | 29.4 | 67,774,943 | 135,046,350 |
| Namibia | 0.6 | 1.0 | 1.7 | 2.4 | 4,339,498 | 5,834,532 |
| Niger | 3.3 | 5.9 | 11.3 | 22.4 | 68,453,727 | 192,186,560 |
| Nigeria | 45.1 | 76.4 | 122.0 | 195.5 | 410,637,868 | 793,942,316 |
| Rwanda | 2.9 | 5.1 | 7.2 | 12.3 | 21,886,077 | 28,184,816 |
| Senegal | 3.2 | 5.5 | 9.7 | 15.8 | 34,030,620 | 64,806,263 |
| Sierra Leone | 2.3 | 3.3 | 4.5 | 7.6 | 12,971,626 | 16,491,439 |
| South Africa | 17.0 | 28.5 | 44.9 | 57.7 | 72,754,583 | 76,487,698 |
| South Sudan | 2.8 | 4.5 | 6.9 | 10.9 | 25,366,221 | 42,794,448 |
| Sudan | 7.5 | 14.5 | 27.2 | 41.8 | 80,385,607 | 138,648,073 |
| Tanzania | 10.5 | 18.5 | 33.4 | 56.3 | 138,081,621 | 303,831,815 |
| Togo | 1.5 | 2.7 | 3.1 | 5.2 | 15,298,154 | 25,246,548 |
| Tunisia | 4.1 | 6.3 | 9.7 | 11.5 | 13,883,996 | 13,321,053 |
| Uganda | 6.7 | 12.4 | 23.6 | 42.7 | 105,698,201 | 213,758,214 |
| Zambia | 3.0 | 5.8 | 10.4 | 17.3 | 41,000,822 | 94,409,602 |
| Zimbabwe | 3.7 | 7.4 | 11.8 | 14.4 | 29,658,750 | 40,687,107 |
| Eritrea | 1.0 | 1.7 | 2.2. | 3.2 | 9,606,664 | 14,781,199 |
| **Total** | **274.4** | **462.3** | **779.9** | **1,232.7** | **2,463,732,808** | **4,362,541,631** |
|  |  |  |  |  |  |  |
| **Madagascar** | **5.0** | **8.7** | **15.7** | **26.2** | **53,802,574** | **98,001,584** |
|  |  |  |  |  |  |  |
| **Neotropics** |  |  |  |  |  |  |
| Belize | 0.092 | 0.140 | 0.247 | 0.383 | 592,420 | 687,935 |
| Costa Rica | 1.3 | 2.3 | 3.9 | 4.9 | 5,774,070 | 5,027,693 |
| El Salvador | 2.7 | 4.5 | 5.8 | 6.4 | 6,997,410 | 5,404,065 |
| Guatemala | 4.2 | 7.2 | 11.6 | 17.2 | 26,968,287 | 32,020,390 |
| Honduras | 2.0 | 3.6 | 11.6 | 17.2 | 13,248,655 | 13,437,227 |
| Mexico | 37.0 | 67.0 | 98.8 | 126.0 | 164,279,302 | 151,491,044 |
| Nicaragua | 1.7 | 3.2 | 5.0 | 6.4 | 7,875,742 | 7,048,824 |
| Panama | 1.1 | 1.9 | 3.0 | 4.1 | 5,827,106 | 6,539,833 |
| Argentina | 20.0 | 27.8 | 36.8 | 44.4 | 55,228,902 | 57,747,478 |
| Bolivia | 3.6 | 5.5 | 8.4 | 11.3 | 15,903,460 | 17,946,304 |
| Brazil | 72.0 | 107.0 | 174.7 | 209.4 | 232,688,044 | 190,423,052 |
| Colombia | 16.0 | 26.9 | 36.6 | 49.6 | 54,732,755 | 44,826,403 |
| Ecuador | 4.50 | 7.90 | 12.60 | 17.00 | 22,968,451 | 24,320,580 |
| French Guiana | **NA** | **NA** | **NA** | **NA** | **NA** | **NA** |
| Guyana | 0.57 | 0.78 | 0.75 | 0.78 | 821,906 | 588,127 |
| Paraguay | 1.9 | 3.1 | 5.3 | 6.9 | 8,897,182 | 8,617,215 |
| Peru | 10.0 | 17.5 | 26.4 | 31-9 | 41,620,307 | 40,783,450 |
| Suriname | 0.3 | 0.4 | 0.5 | 0.6 | 648,399 | 576,392 |
| Trinidad | 0.8 | 1.0 | 1.2 | 1.3 | 1,295,069 | 982,905 |
| Venezuela | 8.1 | 15.1 | 24.1 | 28.8 | 41,584,988 | 41,642,146 |
| **Total** | **187,9** | **302.7** | **467.2** | **552.7** | **707, 952,455** | **650,111,063** |
|  |  |  |  |  |  |  |
|  |  |  |  |  |  |  |
| **South Asia** |  |  |  |  |  |  |
| Afghanistan | 8.1 | 13.3 | 20.7 | 37.1 | 61,928,118 | 70,409,629 |
| Bangladesh | 48.0 | 79.6 | 127.6 | 161.3 | 201,926,816 | 173,548,665 |
| Bhutan | 0.223 | 0.436 | 0.591 | 0.754 | 994,203 | 857,736 |
| Yemen | 5.3 | 7.9 | 17.4 | 28.4 | 48,304,040 | 53,535,658 |
| India | 450.0 | 698.0 | 1,005.0 | 1,300.0 | 1,658,978,162 | 1,516,597,380 |
| Nepal | 10.0 | 15.0 | 23.9 | 28.0 | 36,106,578 | 29,317,902 |
| Pakistan | 44.9 | 78.0 | 142.3 | 212.2 | 306,940,443 | 351,942,931 |
| S. Arabia | 4.0 | 9.6 | 20.6 | 36.6 | 45,056,349 | 44,028,699 |
| **Total** | **570.5** | **901.8** | **1,358.1** | **1,804.4** | **2,360,234,709** | **2,240,238,600** |
|  |  |  |  |  |  |  |
| **Southeast Asia** |  |  |  |  |  |  |
| Brunei | 0.810 | 0.193 | 0.333 | 0.428 | 536,723 | 486,608 |
| Cambodia | 5.7 | 6.6 | 12.1 | 16.2 | 22,019,028 | 22,752,616 |
| China | 66.7 | 981.0 | 1,200.0 | 1,300.0 | 1,364,456,723 | 1,020,665,216 |
| Indonesia | 87.7 | 147.4 | 211.5 | 267.6 | 321,550,686 | 306,025,532 |
| Japan | 92.5 | 116.7 | 126.8 | 126.5 | 108,794,446 | 84,532,388 |
| Laos | 2.1 | 3.2 | 5.3 | 7.0 | 9,162,892 | 8,200,027 |
| Malaysia | 8.1 | 13.1 | 23.1 | 31.5 | 41,729,217 | 41,799,363 |
| Myanmar | 21.7 | 34.2 | 46.7 | 53.7 | 62,358,925 | 54,742,635 |
| Philippines | 26.1 | 47.3 | 77.9 | 53.7 | 151,293,435 | 173,059,809 |
| Singapore | 1.6 | 2.4 | 4.0 | 5.6 | 6,574,759 | 5,431,922 |
| Sri Lanka | 9.8 | 15.0 | 18.7 | 21.6 | 20,792,352 | 15,035,749 |
| Taiwan | NA | NA | NA | NA | 22,770,537 | 16,886,866 |
| Thailand | 27.3 | 47.3 | 62.9 | 69.4 | 65,372,345 | 47,535,689 |
| Timor-Leste | 0.5 | 0.6 | 0.9 | 1.2 | 2,420,760 | 3,847,760 |
| Vietnam | 32.6 | 54.2 | 79.9 | 95.5 | 114,629,852 | 107,645,705 |
| **Total** | **383.184** | **1,469.192** | **1,870.117** | **2,049.928** | **2,314,462,680.000** | **1,908,647,885.000** |
