# Supplementary material for: Current and future trends in socio-economic, demographic and governance factors affecting global primate conservation
Source: PeerJ. 2020 Aug 21;8:e9816. doi: 10.7717/peerj.9816 (PMC7444509; doi:10.7717/peerj.9816)
Supplement: Supplemental Information 5 — Source of data: World Bank https://data.worldbank.org/indicator/SP.RUR.TOTL.ZS?locations=DZ-AO-BJ-AR-BW. Consulted March 2020. [file peerj-08-9816-s005.docx]

**Table S4.** Rural and urban population growth between 1960 and 2018 in countries in primate range regions. Source of data: World Bank https://data.worldbank.org/indicator/SP.RUR.TOTL.ZS?locations=DZ-AO-BJ-AR-BW. Consulted March 2020.

|  | **RURAL** | **RURAL** | **RURAL** | **RURAL** | **URBAN** | **URBAN** | **URBAN** | **URBAN** |
| --- | --- | --- | --- | --- | --- | --- | --- | --- |
|  | **1960** | **1980** | **2000** | **2018** | **1960** | **1980** | **2000** | **2018** |
| **Country** | **%** | **%** | **%** | **%** | **%** | **%** | **%** | **%** |
| **mainland Africa** |  |  |  |  |  |  |  |  |
| Algeria | 69 | 56.4 | 41 | 27 | 31 | 43.6 | 59 | 73 |
| Angola | 90.0 | 75.7 | 50 | 34 | 10 | 24.3 | 50 | 66 |
| Benin | 91.0 | 72.6 | 62 | 53 | 9 | 27.4 | 38 | 47 |
| Botswana | 97.0 | 83.5 | 47 | 31 | 3 | 16.5 | 53 | 69 |
| Burkina Faso | 95.0 | 91.1 | 82 | 71 | 5 | 8.9 | 18 | 29 |
| Burundi | 98.0 | 95.6 | 92 | 87 | 2 | 4.4 | 8 | 13 |
| Somalia | 83.0 | 73.2 | 66 | 55 | 17 | 26.8 | 34 | 45 |
| Ethiopia | 96.0 | 89.5 | 85 | 79 | 4 | 10.5 | 15 | 21 |
| Cameroon | 86.0 | 68.0 | 54 | 44 | 14 | 32 | 46 | 56 |
| Central African Rep | 80.0 | 66.1 | 62 | 59 | 20 | 33.9 | 38 | 41 |
| Chad | 93.0 | 81.2 | 78 | 76 | 7 | 18.8 | 22 | 24 |
| Congo | 68.0 | 52.0 | 41 | 33 | 32 | 48 | 59 | 67 |
| Congo DR | 78.0 | 72,9 | 65 | 56 | 22 | 72.9 | 35 | 44 |
| Cote d’Ivoire | 82.0 | 63.1 | 57 | 49 | 18 | 36.9 | 43 | 51 |
| Djibouti | 49.6 | 27.9 | 23.4 | 87 | 50.4 | 72.1 | 76.6 | 13 |
| Egypt | 62.0 | 56.1 | 57 | 57 | 38 | 43.86 | 43 | 43 |
| Equatorial Guinea | 74.0 | 72.1 | 51 | 28 | 26 | 27.9 | 49 | 72 |
| Eswatini | 96.0 | 83.5 | 77 | 76 | 4 | 16.5 | 23 | 24 |
| Gabon | 81.0 | 45.0 | 21 | 11 | 19 | 55 | 79 | 89 |
| Gambia | 88.0 | 71.5 | 52 | 39 | 12 | 28.5 | 48 | 61 |
| Ghana | 77.0 | 69.0 | 56 | 44 | 23 | 31 | 44 | 56 |
| Guinea | 89.0 | 76.3 | 69 | 64 | 11 | 23.7 | 31 | 36 |
| Guinea-Bissau | 86.0 | 82.1 | 64 | 57 | 14 | 17.9 | 36 | 43 |
| Kenya | 93.0 | 84.4 | 80 | 73 | 7 | 15.6 | 20 | 27 |
| Lesotho | 96.0 | 88.5 | 80 | 72 | 4 | 11.5 | 20 | 28 |
| Liberia | 81.0 | 64.8 | 55 | 49 | 19 | 35.2 | 45 | 51 |
| Malawi | 96.0 | 90.9 | 85 | 83 | 4 | 9.1 | 15 | 17 |
| Mali | 89.0 | 81.5 | 72 | 58 | 11 | 18.5 | 28 | 42 |
| Mauritania | 93.0 | 72.6 | 61 | 47 | 7 | 27.4 | 39 | 53 |
| Morocco | 71.0 | 58.7 | 47 | 37 | 29 | 41.3 | 53 | 63 |
| Mozambique | 93.0 | 86.8 | 71 | 64 | 7 | 13.2 | 29 | 36 |
| Namibia | 82.0 | 74.9 | 67 | 50 | 18 | 25.1 | 33 | 50 |
| Niger | 94.0 | 86.5 | 84 | 84 | 6 | 13.5 | 16 | 16 |
| Nigeria | 84.0 | 78.0 | 65 | 50 | 16 | 22 | 35 | 50 |
| Rwanda | 97.0 | 95.2 | 85 | 83 | 3 | 4.8 | 15 | 17 |
| Senegal | 77.0 | 64.2 | 60 | 53 | 23 | 35.8 | 40 | 47 |
| Sierra Leone | 83.0 | 70.1 | 64 | 58 | 17 | 29.9 | 36 | 42 |
| South Africa | 53.0 | 51.5 | 43 | 33 | 47 | 48.5 | 57 | 67 |
| South Sudan | 91.0 | 91.4 | 83 | 80 | 9 | 8.6 | 17 | 20 |
| Sudan | 89.0 | 80.0 | 67 | 65 | 11 | 20 | 33 | 35 |
| Tanzania | 95.0 | 85.4 | 78 | 67 | 5 | 14.6 | 22 | 33 |
| Togo | 90.0 | 75.3 | 67 | 58 | 10 | 24.7 | 33 | 42 |
| Tunisia | 61.0 | 49.4 | 36 | 31 | 39 | 50.6 | 64 | 69 |
| Uganda | 95.0 | 92.4 | 75 | 86 | 5 | 7.6 | 25 | 14 |
| Zambia | 81.0 | 60.1 | 65 | 56 | 19 | 39.9 | 35 | 44 |
| Zimbabwe | 87.0 | 77.6 | 66 | 66 | 13 | 22.4 | 34 | 34 |
| Eritrea | 90.0 | 85.6 | 73 | 59 | 10 | 14.4 | 27 | 41 |
|  |  |  |  |  |  |  |  |  |
| **Madagascar** | 89.0 | 81.4 | 73 | 63 | 11 | 19 | 27 | 37 |
|  |  |  |  |  |  |  |  |  |
| **Neotropics** |  |  |  |  |  |  |  |  |
| Belize | 45 | 51 | 52 | 54 | 55 | 49.4 | 48 | 46 |
| Costa Rica | 66.0 | 56.9 | 41 | 21 | 34 | 43.1 | 59 | 79 |
| El Salvador | 62.0 | 55.9 | 41 | 28 | 38 | 44.1 | 59 | 72 |
| Guatemala | 69.0 | 61.2 | 55 | 49 | 31 | 38.8 | 45 | 51 |
| Honduras | 77.0 | 65.1 | 55 | 43 | 23 | 34.9 | 45 | 57 |
| Mexico | 49.0 | 33.6 | 25 | 20 | 51 | 66.4 | 75 | 80 |
| Nicaragua | 60.0 | 33.6 | 45 | 41 | 40 | 66.4 | 55 | 59 |
| Panama | 59.0 | 49.7 | 38 | 32 | 41 | 50.3 | 62 | 68 |
| Argentina | 26.0 | 17.1 | 11 | 8 | 74 | 82.9 | 89 | 92 |
| Bolivia | 63.0 | 54.5 | 39 | 31 | 37 | 45.5 | 61 | 69 |
| Brazil | 54.0 | 34.5 | 19 | 13 | 46 | 65.5 | 81 | 87 |
| Colombia | 54.0 | 36.2 | 26 | 18 | 46 | 63.8 | 74 | 82 |
| Ecuador | 66.0 | 53.0 | 40 | 36 | 34 | 47 | 60 | 64 |
| French Guiana | **NA** | **NA** | **NA** | **NA** | **NA** | **NA** | **NA** | **NA** |
| Guyana | 71.0 | 69.5 | 71 | 73 | 29 | 30.5 | 29 | 27 |
| Paraguay | 64.0 | 58.3 | 45 | 38 | 36 | 41.7 | 55 | 62 |
| Peru | 53.0 | 35.4 | 27 | 22 | 47 | 64.6 | 73 | 78 |
| Suriname | 53.0 | 35.0 | 34 | 34 | 47 | 65 | 66 | 66 |
| Trinidad | 66.0 | 47.7 | 44 | 47 | 34 | 52.3 | 56 | 53 |
| Venezuela | 38.0 | 20.8 | 12 | 12 | 62 | 79.2 | 88 | 88 |
|  |  |  |  |  |  |  |  |  |
|  |  |  |  |  |  |  |  |  |
| **South Asia** |  |  |  |  |  |  |  |  |
| Afghanistan | 91.0 | 84.0 | 78 | 74.5 | 9 | 16 | 22 | 25.5 |
| Bangladesh | 95.0 | 85.1 | 76 | 65 | 5 | 14.9 | 24 | 35 |
| Bhutan | 96.0 | 89.8 | 74 | 59 | 4 | 10.2 | 26 | 41 |
| Yemen | 91.0 | 83.4 | 73 | 63 | 9 | 16.6 | 27 | 37 |
| India | 82.0 | 76.9 | 72 | 66 | 18 | 23.1 | 28 | 34 |
| Nepal | 96.0 | 93.9 | 86 | 80 | 4 | 6.1 | 14 | 20 |
| Pakistan | 78.0 | 71.9 | 67 | 63 | 22 | 28.1 | 33 | 37 |
| S. Arabia | 69.0 | 34.1 | 20 | 16 | 31 | 65.9 | 80 | 84 |
|  |  |  |  |  |  |  |  |  |
| **Southeast Asia** |  |  |  |  |  |  |  |  |
| Brunei | 57.0 | 35.0 | 29 | 22 | 43 | 65 | 71 | 78 |
| Cambodia | 90.0 | 90.0 | 81 | 76 | 10 | 10 | 19 | 24 |
| China | 84.0 | 80.6 | 64 | 40 | 16 | 19.4 | 36 | 60 |
| Indonesia | 85.0 | 77.8 | 58 | 45 | 15 | 22.2 | 42 | 55 |
| Japan | 37.0 | 23.8 | 21 | 8 | 63 | 76.2 | 79 | 92 |
| Laos | 92.0 | 87.6 | 78 | 65 | 8 | 12.4 | 22 | 35 |
| Malaysia | 73.0 | 57.9 | 38 | 24 | 27 | 42.1 | 62 | 76 |
| Myanmar | 80.0 | 76.0 | 73 | 69 | 20 | 24 | 27 | 31 |
| Philippines | 70.0 | 62.5 | 53 | 53 | 30 | 37.5 | 47 | 47 |
| Singapore | NA | NA | NA | NA | NA | NA | NA | NA |
| Sri Lanka | 83.0 | 81.0 | 81 | 81 | 17 | 19 | 19 | 19 |
| Taiwan | NA | NA | NA | NA | NA | NA | NA | NA |
| Thailand | 80.0 | 73.2 | 68 | 50 | 20 | 26.8 | 32 | 50 |
| Timor-Leste | 90.0 | 83.5 | 75 | 69 | 10 | 16.5 | 25 | 31 |
| Vietnam | 85.0 | 80.7 | 75 | 64 | 15 | 19.3 | 25 | 36 |
