# Supplementary material for: Current and future trends in socio-economic, demographic and governance factors affecting global primate conservation
Source: PeerJ. 2020 Aug 21;8:e9816. doi: 10.7717/peerj.9816 (PMC7444509; doi:10.7717/peerj.9816)
Supplement: Supplemental Information 6 — 2018 Gross Domestic Product per Capita (GDDPC), 2018 Human Development Index, 2019 Global Food Security Index, and 2019 Corruption Perception Index (CPI) for primate range countries. NA = not available. Sources of data for GDPPC, The World Bank https://data.worldbank.org/indicator/NY.GDP.PCAP.CD. Human Development Index, United Nations Development Programme http://hdr.undp.org/en/data, http://hdr.undp.org/en/data. Global Food Security Index, Economist Intelligent Unit https://foodsecurityindex.eiu.com/Index. Corruption Perception Index, Transparency International www.transparency.org/cpi. Each of these sources was consulted in March 2020. [file peerj-08-9816-s006.docx]

**Table S5.** 2018 Gross Domestic Product per Capita (GDDPC), 2018 Human Development Index, 2019 Global Food Security Index, and 2019 Corruption Perception Index for primate range countries. NA = not available. Sources of data for GDPPC, The World Bank https://data.worldbank.org/indicator/NY.GDP.PCAP.CD. Human Development Index, United Nations Development Programme http://hdr.undp.org/en/data, http://hdr.undp.org/en/data. Global Food Security Index, Economist Intelligent Unit https://foodsecurityindex.eiu.com/Index. Corruption Perception Index, Transparency International www.transparency.org/cpi. Each of these sources was consulted in March 2020.

|  | **Gross Domestic Product per Capita (CDPPC)** | **Human Development Index (HDI)** | **Global Food Security Index 2019 (GFSI)** | **Corruption Perception Index 2019 (CPI)** |
| --- | --- | --- | --- | --- |
| **Country** | **US$** | **0 low - 1.0 high** | **0 low – 100 high** | **0 highly corrupt to 100 very clean** |
| **mainland Africa** |  |  |  |  |
| Algeria | 4,114.70 | 0.759 | 59.8 | 35 |
| Angola | 3,432.40 | 0.574 | 45.5 | 26 |
| Benin | 901.5 | 0.520 | 51 | 41 |
| Botswana | 8,258.60 | 0.728 | 63.8 | 61 |
| Burkina Faso | 715.1 | 0.434 | 50.1 | 40 |
| Burundi | 271.8 | 0.423 | 34.3 | 19 |
| Somalia | 314.5 | NA | NA | NA |
| Ethiopia | 772.3 | 0.470 | 49.2 | 37 |
| Cameroon | 1,533.70 | 0.563 | 48.9 | 25 |
| Central African Republic | 475.7 | 0.381 | NA | 25 |
| Chad | 728.3 | 0.401 | 36.9 | NA |
| Congo | 2,147.80 | 0.608 | NA | 19 |
| Congo DR | 561.8 | 0.459 | 35.7 | 18 |
| Cote d’Ivoire | 1,715.50 | 0.516 | 52.3 | 35 |
| Djibouti | 3,082.50 | 0.495 | NA | 30 |
| Egypt | 2,549.10 | 0.700 | 64.5 | 35 |
| Equatorial Guinea | 10,261.80 | 0.588 | NA | 16 |
| Eswatini | 4,146.00 | 0.608 | NA | 34 |
| Gabon | 7,952.50 | 0.702 | NA | 31 |
| Gambia | 716.1 | 0.466 | NA | 37 |
| Ghana | 2,202.30 | 0.596 | 62.8 | 41 |
| Guinea | 878.6 | 0.466 | 46.7 | 29 |
| Guinea-Bissau | 778 | 0.461 | NA | 18 |
| Kenya | 1,710.50 | 0.579 | 50.7 | 28 |
| Lesotho | 1,299.20 | 0.518 | NA | 40 |
| Liberia | 677.3 | 0.465 | NA | 28 |
| Malawi | 389.4 | 0.485 | 42.5 | 31 |
| Mali | 899.7 | 0.427 | 54.4 | 29 |
| Mauritania | 1,188.80 | NA | NA | 28 |
| Morocco | 3,237.90 | 0.676 | 62.8 | 41 |
| Mozambique | 499 | 0.446 | 41.4 | 26 |
| Namibia | 5,931.50 | 0.645 | NA | 52 |
| Niger | 414 | 0.377 | 49.6 | 32 |
| Nigeria | 2,028.20 | 0.534 | 48.4 | 26 |
| Rwanda | 772.9 | 0.536 | 48.2 | 53 |
| Senegal | 1,522.00 | NA | 54.3 | 45 |
| Sierra Leone | 534 | 0.438 | 39 | 33 |
| South Africa | 6,374.00 | 0.705 | 67.3 | 44 |
| South Sudan | 1,119.70 | 0.413 | NA | 12 |
| Sudan | 977.3 | 0.507 | 45.7 | 16 |
| Tanzania | 1,050.70 | 0.528 | 47.6 | 37 |
| Togo | 679.3 | 0.513 | 44 | 29 |
| Tunisia | 3,447.50 | 0.739 | 60.1 | NA |
| Uganda | 642.8 | 0.528 | 46.2 | 28 |
| Zambia | 1,539.90 | 0.591 | 44.4 | 34 |
| Zimbabwe | 2,147.00 | 0.563 | NA | 24 |
| Eritrea | 811.4 | 0.434 | NA | A |
|  |  |  |  |  |
| **Madagascar** | 527.5 | 0.521 | 37.9 | 24 |
|  |  |  |  |  |
| **Neotropics** |  |  |  |  |
| Belize | 4,884.70 | 0.720 | NA | NA |
| Costa Rica | 12,027.40 | 0.794 | 70.1 | 44 |
| El Salvador | 4,058.30 | 0.667 | 60.7 | 34 |
| Guatemala | 4,549.00 | 0.651 | 60.6 | 26 |
| Honduras | 2,500.10 | 0.623 | 58 | 26 |
| Mexico | 9,673.40 | 0.767 | 69.4 | 29 |
| Nicaragua | 2,028.90 | 0.651 | 54.2 | 22 |
| Panama | 15,575.10 | 0.795 | 68.8 | 36 |
| Argentina | 11,684 | 0.830 | 70.8 | 45 |
| Bolivia | 3,548.60 | 0.703 | 57.7 | 31 |
| Brazil | 8,920.80 | 0.761 | 70.1 | 35 |
| Colombia | 6,667.80 | 0.761 | 69.4 | 37 |
| Ecuador | 6,344.90 | 0.758 | 61.8 | 38 |
| French Guiana | 18,300 | 0.67 | NA | NA |
| Guyana | 868.3 | 0.670 | NA | 40 |
| Paraguay | 5,821.80 | 0.724 | 57.9 | 28 |
| Peru | 6,941.20 | 0.759 | 63.3 | 36 |
| Suriname | 6,234.00 | 0.724 | NA | 44 |
| Trinidad | 17,129.90 | 0.799 | NA | 40 |
| Venezuela | 16,054.50 | 0.726 | 31.2 | 16 |
|  |  |  |  |  |
| **South Asia** |  |  |  |  |
| Afghanistan | 520.9 | 0.496 | NA | 16 |
| Bangladesh | 1,698.30 | 0.614 | 53.2 | 26 |
| Bhutan | 3,360.30 | 0.617 | NA | 68 |
| Yemen | 944.4 | 0.463 | NA | 15 |
| India | 2,010.00 | 0.647 | 58.9 | 41 |
| Nepal | 1,033.90 | 0.579 | 56.4 | 34 |
| Pakistan | 1,482.40 | 0.560 | 56.8 | 32 |
| Saudi Arabia | 23,339.00 | 0.857 | NA | 53 |
|  |  |  |  |  |
| **Southeast Asia** |  |  |  |  |
| Brunei | 31,628.30 | 0.845 | NA | 60 |
| Cambodia | 1,510.30 | 0.581 | 49.4 | 20 |
| China | 9,770.80 | 0.758 | 71 | 41 |
| Indonesia | 9,770.80 | 0.707 | 62.6 | 40 |
| Japan | 40,847.00 | 0.915 | 76.5 | NA |
| Lao PDR | 2,542.50 | 0.604 | 49.1 | 29 |
| Malaysia | 11,373.20 | 0.804 | 73.8 | 53 |
| Myanmar | 1,326.00 | 0.584 | 57 | 29 |
| Philippines | 3,102.70 | 0.712 | 61 | 34 |
| Singapore | 64,581.90 | 0.935 | 87.4 | 85 |
| Sri Lanka | 4,102.50 | 0.780 | 60.8 | 38 |
| Taiwan | 24,827.90 | 0.882 | NA | 65 |
| Thailand | 7,273.60 | 0.765 | 65.1 | 36 |
| Timor-Leste | 2,035.50 | 0.626 | NA | 38 |
| Vietnam | 2,566.60 | 0.693 | 64.6 | 37 |
