# Supplementary material for: Current and future trends in socio-economic, demographic and governance factors affecting global primate conservation
Source: PeerJ. 2020 Aug 21;8:e9816. doi: 10.7717/peerj.9816 (PMC7444509; doi:10.7717/peerj.9816)
Supplement: Supplemental Information 7 — UN Human Development Index (HDI: 0 low, 1.0 highest). United Nations Development Programme. http://hdr.undp.org/en/data; http://worldpopulationreview.com/countries/hdi-by-country/ Consulted March 2020. [file peerj-08-9816-s007.docx]

**Table S6.** UN Human Development Index (HDI: 0 low, 1.0 highest). United Nations Development Programme. http://hdr.undp.org/en/data; <http://worldpopulationreview.com/countries/hdi-by-country/> Consulted March 2020.

|  |  |  |  |  |
| --- | --- | --- | --- | --- |
|  |  | **HDI** |  |  |
| Country | **1990** | **2000** | **2010** | **2018** |
|  |  |  |  |  |
| **mainland Africa** |  |  |  |  |
| Algeria | 0.58 | 0.65 | 0.73 | 0.76 |
| Angola | … | 0.39 | 0.51 | 0.57 |
| Benin | 0.35 | 0.40 | 0.47 | 0.52 |
| Botswana | 0.57 | 0.58 | 0.66 | 0.73 |
| Burkina Faso | .. | 0.29 | 0.38 | 0.43 |
| Burundi | 0.30 | 0.29 | 0.40 | 0.42 |
| Somalia | .. | .. | .. | .. |
| Ethiopia | .. | .. | .. | .. |
| Cameroon | 0.45 | 0.44 | 0.47 | 0.56 |
| Central African Republic | 0.32 | 0.31 | 0.36 | 0.38 |
| Chad | .. | 0.30 | 0.37 | 0.40 |
| Congo | 0.53 | 0.50 | 0.56 | 0.61 |
| Congo DR | 0.38 | 0.33 | 0.42 | 0.46 |
| Cote d’Ivoire | 0.39 | 0.41 | 0.45 | 0.52 |
| Djibouti | .. | 0.36 | 0.45 | 0.50 |
| Egypt | 0.55 | 0.61 | 0.67 | 0.70 |
| Equatorial Guinea | .. | 0.52 | 0.58 | 0.59 |
| Eswatini | 0.55 | 0.47 | 0.51 | 0.61 |
| Gabon | 0.62 | 0.63 | 0.66 | 0.70 |
| Gambia | 0.33 | 0.38 | 0.44 | 0.47 |
| Ghana | 0.45 | 0.48 | 0.55 | 0.60 |
| Guinea | 0.28 | 0.34 | 0.41 | 0.47 |
| Guinea-Bissau | .. | .. | 0.43 | 0.46 |
| Kenya | 0.47 | 0.45 | 0.53 | 0.58 |
| Lesotho | 0.49 | 0.44 | 0.46 | 0.52 |
| Liberia | .. | 0.42 | 0.44 | 0.47 |
| Malawi | 0.30 | 0.36 | 0.44 | 0.49 |
| Mali | 0.23 | 0.31 | 0.40 | 0.43 |
| Mauritania | 0.38 | 0.45 | 0.49 | 0.53 |
| Morocco | 0.46 | 0.53 | 0.62 | 0.68 |
| Mozambique | 0.22 | 0.30 | 0.40 | 0.45 |
| Namibia | 0.58 | 0.54 | 0.59 | 0.65 |
| Niger | 0.21 | 0.25 | 0.32 | 0.38 |
| Nigeria | .. | .. | 0.48 | 0.53 |
| Rwanda | 0.25 | 0.34 | 0.49 | 0.54 |
| Senegal | 0.38 | 0.39 | 0.47 | 0.51 |
| Sierra Leone | 0.27 | 0.30 | 0.39 | 0.44 |
| South Africa | 0.63 | 0.63 | 0.66 | 0.71 |
| South Sudan | .. | .. | 0.43 | 0.41 |
| Sudan | 0.33 | 0.40 | 0.47 | 0.51 |
| Tanzania | 0.37 | 0.40 | 0.49 | 0.53 |
| Togo | 0.41 | 0.43 | 0.47 | 0.51 |
| Tunisia | 0.57 | 0.65 | 0.72 | 0.74 |
| Uganda | 0.31 | 0.40 | 0.49 | 0.53 |
| Zambia | 0.42 | 0.43 | 0.53 | 0.59 |
| Zimbabwe | 0.50 | 0.45 | 0.47 | 0.56 |
| Eritrea | … | … | … | … |
| **AVG** | **0.41** | **0.43** | **0.49** | **0.54** |
|  |  |  |  |  |
| **Madagascar** | **…** | **0.46** | **0.50** | **0.52** |
|  |  |  |  |  |
| **Neotropics** |  |  |  |  |
| Belize | 0.61 | 0.64 | 0.69 | 0.72 |
| Costa Rica | 0.66 | 0.71 | 0.75 | 0.79 |
| El Salvador | 0.53 | 0.61 | 0.66 | 0.67 |
| Guatemala | 0.48 | 0.55 | 0.60 | 0.65 |
| Honduras | 0.51 | 0.56 | 0.60 | 0.62 |
| Mexico | 0.65 | 0.71 | 0.74 | 0.77 |
| Nicaragua | 0.49 | 0.57 | 0.61 | 0.65 |
| Panama | 0.66 | 0.72 | 0.76 | 0.80 |
| Argentina | 0.71 | 0.77 | 0.82 | 0.83 |
| Bolivia | 0.54 | 0.62 | 0.66 | 0.70 |
| Brazil | 0.61 | 0.68 | 0.73 | 0.76 |
| Colombia | 0.60 | 0.66 | 0.73 | 0.76 |
| Ecuador | 0.64 | 0.67 | 0.72 | 0.76 |
| French Guiana |  |  |  |  |
| Guyana | 0.54 | 0.61 | 0.64 | 0.67 |
| Paraguay | 0.59 | 0.64 | 0.69 | 0.72 |
| Peru | 0.61 | 0.68 | 0.72 | 0.76 |
| Suriname |  |  |  |  |
| Trinidad | 0.67 | 0.72 | 0.79 | 0.80 |
| Venezuela | 0.64 | 0.67 | 0.75 | 0.73 |
|  |  |  |  |  |
| **Avg Neotropics** | **0.60** | **0.65** | **0.70** | **0.73** |
|  |  |  |  |  |
| **South Asia** |  |  |  |  |
| Afghanistan | 0.30 | 0.35 | 0.46 | 0.50 |
| Bangladesh |  |  |  |  |
| Bhutan | .. | .. | 0.57 | 0.62 |
| Yemen | 0.39 | 0.43 | 0.50 | 0.46 |
| India | 0.43 | 0.50 | 0.58 | 0.65 |
| Nepal | 0.38 | 0.45 | 0.53 | 0.58 |
| Pakistan | 0.40 | 0.45 | 0.52 | 0.56 |
| Saudi Arabia | 0.70 | 0.74 | 0.81 | 0.86 |
| **AVG** | **0.43** | **0.49** | **0.57** | **0.60** |
|  |  |  |  |  |
| **Southeast Asia** |  |  |  |  |
| Brunei | 0.77 | 0.81 | 0.83 | 0.85 |
| Cambodia | 0.38 | 0.42 | 0.54 | 0.58 |
| China | 0.50 | 0.59 | 0.70 | 0.76 |
| Indonesia | 0.53 | 0.60 | 0.67 | 0.71 |
| Japan | 0.82 | 0.86 | 0.89 | 0.92 |
| Lao PDR | 0.40 | 0.47 | 0.55 | 0.60 |
| Malaysia | 0.64 | 0.72 | 0.77 | 0.80 |
| Myanmar | 0.35 | 0.42 | 0.52 | 0.58 |
| Philippines | 0.59 | 0.63 | 0.67 | 0.71 |
| Singapore | 0.72 | 0.82 | 0.91 | 0.94 |
| Sri Lanka | 0.63 | 0.69 | 0.75 | 0.78 |
| Taiwan | … | … | … | … |
| Thailand | 0.57 | 0.65 | 0.72 | 0.77 |
| Timor-Leste | .. | 0.51 | 0.62 | 0.63 |
| Vietnam | 0.48 | 0.58 | 0.65 | 0.69 |
| **AVG** | **0.57** | **0.63** | **0.70** | **0.74** |
|  |  |  |  |  |
| **World avg** | **0.60** | **0.64** | **0.70** | **0.73** |
|  |  |  |  |  |
| **Top 25 developed nations** |  |  |  |  |
| **Country** | **1990** | **2000** | **2010** | **2018** |
| Norway | 0.85 | 0.917 | 0.942 | 0.954 |
| Switzerland | 0.832 | 0.889 | 0.932 | 0.946 |
| Ireland | 0.764 | 0.857 | 0.89 | 0.942 |
| Germany | 0.801 | 0.869 | 0.92 | 0.939 |
| Hong Kong, China (SAR) | 0.781 | 0.827 | 0.901 | 0.939 |
| Australia | 0.866 | 0.898 | 0.926 | 0.938 |
| Iceland | 0.804 | 0.861 | 0.892 | 0.938 |
| Sweden | 0.816 | 0.897 | 0.906 | 0.937 |
| Singapore | 0.718 | 0.818 | 0.909 | 0.935 |
| Netherlands | 0.83 | 0.876 | 0.911 | 0.934 |
| Denmark | 0.799 | 0.863 | 0.91 | 0.93 |
| Finland | 0.784 | 0.858 | 0.903 | 0.925 |
| Canada | 0.85 | 0.868 | 0.895 | 0.922 |
| New Zealand | 0.82 | 0.87 | 0.899 | 0.921 |
| United Kingdom | 0.775 | 0.867 | 0.905 | 0.92 |
| United States | 0.86 | 0.881 | 0.911 | 0.92 |
| Belgium | 0.806 | 0.873 | 0.903 | 0.919 |
| Liechtenstein | .. | 0.862 | 0.904 | 0.917 |
| Japan | 0.816 | 0.855 | 0.885 | 0.915 |
| Austria | 0.795 | 0.838 | 0.895 | 0.914 |
| Luxembourg | 0.791 | 0.86 | 0.893 | 0.909 |
| Israel | 0.792 | 0.853 | 0.887 | 0.906 |
| Korea (Republic of) | 0.728 | 0.817 | 0.882 | 0.906 |
| Slovenia | 0.829 | 0.824 | 0.881 | 0.902 |
| Spain | 0.754 | 0.825 | 0.865 | 0.893 |
| **AVG** | **0.803** | **0.861** | **0.902** | **0.925** |
