# Supplementary material for: Current and future trends in socio-economic, demographic and governance factors affecting global primate conservation
Source: PeerJ. 2020 Aug 21;8:e9816. doi: 10.7717/peerj.9816 (PMC7444509; doi:10.7717/peerj.9816)
Supplement: Supplemental Information 8 — 2018 Under-five mortality. Source World Bank https://data.worldbank.org/indicator/SH.DTH.MORT Consulted March 2020. Summaries are shown for primate regions, for top 25 developed nations and for the EU, UK, USA and Canada. [file peerj-08-9816-s008.docx]

**Table S7.** 2018 Under-five mortality. Source World Bank <https://data.worldbank.org/indicator/SH.DTH.MORT>

Consulted March 2020. Summaries are shown for primate regions, for top 25 developed nations and for the EU, UK, USA and Canada.

| **Primate range** | **2018 Under-five deaths** |  |  |  |
| --- | --- | --- | --- | --- |
| Africa (48) | 2,867,673 |  |  |  |
| South Asia (8) | 1,474,625 |  |  |  |
| Southeast Asia (15) | 462,367 |  |  |  |
| Neotropics (20) | 172,316 |  |  |  |
|  |  |  |  |  |
| EU (28), UK,USA, CAN | 51,000 |  |  |  |
|  |  |  |  |  |
|  |  |  | **2018 Under -five mortality** |  |
|  |  |  | **USA** | 25,497 |
| **Top developed nations** | **2018 Under five mortality** |  | **European Union (n = 28)** | 19,853 |
| Luxembourg | 15 |  | **UK** | 3,316 |
| Norway | 150 |  | **Canada** | 1,919 |
| Switzerland | 360 |  | **Total EU, UK, Canada** | **50,585** |
| Qatar | 179 |  |  |  |
| Ireland | 232 |  |  |  |
| Denmark | 257 |  |  |  |
| Sweden | 320 |  |  |  |
| Australia | 1171 |  |  |  |
| US | 25,497 |  |  |  |
| Netherlands | 668 |  |  |  |
| Canada | 1,919 |  |  |  |
| Austria | 306 |  |  |  |
| Japan | 2,424 |  |  |  |
| Iceland | 8 |  |  |  |
| Germany | 2,807 |  |  |  |
| Finland | 89 |  |  |  |
| Belgium | 456 |  |  |  |
| United Kingdom | 3,316 |  |  |  |
| France | 2,980 |  |  |  |
| New Zealand | 341 |  |  |  |
| Italy | 1,417 |  |  |  |
| Spain | 1,219 |  |  |  |
| Korea (Republic of) | 1,237 |  |  |  |
| Greece | 372 |  |  |  |
| Czech Republic | 373 |  |  |  |
