# Supplementary material for: Current and future trends in socio-economic, demographic and governance factors affecting global primate conservation
Source: PeerJ. 2020 Aug 21;8:e9816. doi: 10.7717/peerj.9816 (PMC7444509; doi:10.7717/peerj.9816)
Supplement: Supplemental Information 9 — Global Food Security Index (FSI: 0 low, 100 high) of The Economist Intelligent Unit Limited. https://foodsecurityindex.eiu.com/Index. Consulted March 2020. [file peerj-08-9816-s009.docx]

**Table S8.** Global Food Security Index (**FSI**: 0 low, 100 high) of The Economist Intelligent Unit Limited. https://foodsecurityindex.eiu.com/Index. Consulted March 2020.

|  | **Global Food Security Index** | | |  |
| --- | --- | --- | --- | --- |
|  | <https://foodsecurityindex.eiu.com/Index> | | | |
|  |  |  |  |  |
|  | **0 low FSI, 100 high FSI** | | |  |
| **Country** | **2019 FSI** |  | **Country** | **2019 FSI** |
| **mainland Africa** |  |  | **Neotropics** |  |
| Algeria | 59.8 |  | Belize | NA |
| Angola | 45.5 |  | Costa Rica | 70.1 |
| Benin | 51 |  | El Salvador | 60.7 |
| Botswana | 63.8 |  | Guatemala | 60.6 |
| Burkina Faso | 50.1 |  | Honduras | 58 |
| Burundi | 34.3 |  | Mexico | 69.4 |
| Ethiopia | NA |  | Nicaragua | 54.2 |
| Somalia | NA |  | Panama | 68.8 |
| Cameroon | 49.9 |  | Argentina | 70.8 |
| Central African Republic | NA |  | Bolivia | 57.7 |
| Chad | 36.9 |  | Brazil | 70.1 |
| Congo | NA |  | Colombia | 69.4 |
| Congo DR | 35.7 |  | Ecuador | 61.8 |
| Cote d’Ivoire | 52.3 |  | French Guiana | NA |
| Djibouti | NA |  | Guyana | NA |
| Egypt | 64.5 |  | Paraguay | 57.9 |
| Equatorial Guinea | NA |  | Peru | 63.3 |
| Eswatini | NA |  | Suriname | NA |
| Gabon | NA |  | Trinidad | NA |
| Gambia | NA |  | Venezuela | 31.2 |
| Ghana | NA |  | **AVG** | **61.6** |
| Guinea | 46.7 |  |  |  |
| Guinea-Bissau | NA |  | **South Asia** |  |
| Kenya | 50.7 |  | Afghanistan | NA |
| Lesotho | NA |  | Bangladesh | 53.2 |
| Liberia | NA |  | Bhutan | NA |
| Malawi | 42.5 |  | India | 58.9 |
| Mali | 54.4 |  | Nepal | 56.4 |
| Mauritania | NA |  | Pakistan | 56.8 |
| Morocco | 62.8 |  | Saudi Arabia | 73.5 |
| Mozambique | 41.4 |  | Yemen | 35.6 |
| Namibia | NA |  | **AVG** | **55.7** |
| Niger | 49.6 |  |  |  |
| Nigeria | 48.4 |  | **Southeast Asia** |  |
| Rwanda | 48.2 |  | Brunei | NA |
| Senegal | 54.3 |  | Cambodia | 49.4 |
| Sierra Leone | 39 |  | China | 71 |
| South Africa | 67.3 |  | Indonesia | 62.6 |
| South Sudan | NA |  | Japan | 76.5 |
| Sudan | 45.7 |  | Lao PDR | 49.1 |
| Tanzania | 47.6 |  | Malaysia | 73.8 |
| Togo | 44 |  | Myanmar | 57 |
| Tunisia | 60.1 |  | Philippines | 61 |
| Uganda | 46.2 |  | Singapore | 87.4 |
| Zambia | 44.4 |  | Sri Lanka | 60.8 |
| Zimbabwe | NA |  | Taiwan | NA |
| Eritrea | NA |  | Thailand | 65.1 |
| **AVG** | **49.6** |  | Timor-Leste | NA |
|  |  |  | Vietnam | 64.6 |
| **Madagascar** | **37.9** |  | **AVG** | **64.9** |
