# Supplementary material for: Current and future trends in socio-economic, demographic and governance factors affecting global primate conservation
Source: PeerJ. 2020 Aug 21;8:e9816. doi: 10.7717/peerj.9816 (PMC7444509; doi:10.7717/peerj.9816)
Supplement: Supplemental Information 10 — Perception Corruption Index (PCI: 0 highly corrupt, 100 very clean) of Transparency International www.transparency.org/cpi. Consulted March 2020. The Corruption Perceptions Index is a composite index, a combination of different international surveys and assessments of corruption, collected by a variety of reputable institutions. The index draws on 13 surveys from independent institutions specializing in governance and business climate analysis covering expert assessments and views of businesspeople. None of these surveys were commissioned by Transparency International (https://www.transparency.org/news/pressrelease/explanation_of_how_individual_country_scores_of_the_corruption_perceptions). [file peerj-08-9816-s010.docx]

**Table S9**. **Perception Corruption Index** (**PCI**: 0 highly corrupt, 100 very clean) of Transparency International www.transparency.org/cpi. Consulted March 2020. The Corruption Perceptions Index is a composite index, a combination of different international surveys and assessments of corruption, collected by a variety of reputable institutions. The index draws on 13 surveys from independent institutions specializing in governance and business climate analysis covering expert assessments and views of businesspeople. None of these surveys were commissioned by Transparency International (https://www.transparency.org/news/pressrelease/explanation_of_how_individual_country_scores_of_the_corruption_perceptions).

| **mainland Africa** | 2019 PCI |  | **mainland Africa** | 2019 PCI |
| --- | --- | --- | --- | --- |
| Somalia | 9 |  | Tanzania | 37 |
| South Sudan | 12 |  | Ethiopia | 37 |
| Equatorial Guinea | 16 |  | Burkina Faso | 40 |
| Sudan | 16 |  | Lesotho | 40 |
| Congo DR | 18 |  | Benin | 41 |
| Guinea-Bissau | 18 |  | Ghana | 41 |
| Burundi | 19 |  | Morocco | 41 |
| Republic of Congo | 19 |  | Tunisia | 43 |
| Chad | 20 |  | South Africa | 44 |
| Eritrea | 23 |  | Senegal | 45 |
| Zimbabwe | 24 |  | Namibia | 52 |
| Cameroon | 25 |  | Rwanda | 53 |
| Central African Republic | 25 |  | Botswana | 61 |
| Angola | 26 |  |  |  |
| Mozambique | 26 |  | **Madagascar** | 24 |
| Nigeria | 26 |  |  |  |
| Kenya | 28 |  |  |  |
| Liberia | 28 |  |  |  |
| Mauritania | 28 |  |  |  |
| Uganda | 28 |  |  |  |
| Guinea | 29 |  |  |  |
| Mali | 29 |  |  |  |
| Togo | 29 |  |  |  |
| Djibouti | 30 |  |  |  |
| Gabon | 31 |  |  |  |
| Malawi | 31 |  |  |  |
| Niger | 32 |  |  |  |
| Sierra Leone | 33 |  |  |  |
| Eswatini | 34 |  |  |  |
| Zambia | 34 |  |  |  |
| Algeria | 35 |  |  |  |
| Cote d’Ivoire | 35 |  |  |  |
| Egypt | 35 |  |  |  |
| Gambia | 37 |  |  |  |

**Table S9, continued**. Perception Corruption Index (PCI: 0 highly corrupt, 100 very clean) of Transparency International www.transparency.org/cpi. Consulted March 2020

| **Neotropics** | 2019 PCI |  | **Southeast Asia** | 2019 PCI |  | **Top developed** | 2019 PCI |
| --- | --- | --- | --- | --- | --- | --- | --- |
| Belize | NA |  | Brunei | 60 |  | Denmark | 87 |
| Costa Rica | 56 |  | Cambodia | 20 |  | New Zealand | 87 |
| El Salvador | 34 |  | China | 41 |  | Finland | 86 |
| Guatemala | 26 |  | Indonesia | 40 |  | Switzerland | 85 |
| Honduras | 26 |  | Japan | 73 |  | Sweden | 85 |
| Mexico | 29 |  | Lao PDR | 29 |  | Singapore | 85 |
| Nicaragua | 22 |  | Malaysia | 53 |  | Norway | 84 |
| Panama | 36 |  | Myanmar | 29 |  | Netherlands | 82 |
| Argentina | 45 |  | Philippines | 34 |  | Germany | 80 |
| Bolivia | 31 |  | Singapore | 85 |  | Luxembourg | 80 |
| Brazil | 35 |  | Sri Lanka | 38 |  | Iceland | 78 |
| Colombia | 37 |  | Taiwan | 65 |  | Australia | 77 |
| Ecuador | 38 |  | Thailand | 36 |  | Canada | 77 |
| French Guiana | NA |  | Timor-Leste | 38 |  | United Kingdom | 77 |
| Guyana | 40 |  | Vietnam | 37 |  | Austria | 77 |
| Paraguay | 28 |  |  |  |  | Hong Kong, China (SAR) | 76 |
| Peru | 36 |  |  |  |  | Belgium | 75 |
| Suriname | 44 |  |  |  |  | Ireland | 74 |
| Trinidad | 40 |  |  |  |  | Japan | 73 |
| Venezuela | 16 |  |  |  |  | United Aram Emirates | 71 |
|  |  |  |  |  |  | Uruguay | 71 |
| **South Asia** |  |  |  |  |  | United States | 69 |
| Afghanistan | 16 |  |  |  |  | France | 69 |
| Bangladesh | 26 |  |  |  |  | Spain | 62 |
| Bhutan | 68 |  |  |  |  | Israel | 60 |
| India | 41 |  |  |  |  |  |  |
| Nepal | 34 |  |  |  |  |  |  |
| Pakistan | 32 |  |  |  |  |  |  |
| Saudi Arabia | 53 |  |  |  |  |  |  |
| Yemen | 15 |  |  |  |  |  |  |
