# Supplementary material for: Current and future trends in socio-economic, demographic and governance factors affecting global primate conservation
Source: PeerJ. 2020 Aug 21;8:e9816. doi: 10.7717/peerj.9816 (PMC7444509; doi:10.7717/peerj.9816)
Supplement: Supplemental Information 11 — Global Peace Index (GPI: 1 most peaceful, 5 least peaceful) of the Institute of Economics and Peace. http://economicsandpeace.org/reports/. Global Peace Index 2019 list of countries is from: Measuring Peace in a Complex World, Sydney, June 2019. Available from: http://visionofhumanity.org/reports (Consulted March 2020). [file peerj-08-9816-s011.docx]

**Table S10. Global Peace Index** **(GPI:** 1 most peaceful, 5 least peaceful**)** of the Institute of Economics and Peace. <http://economicsandpeace.org/reports/>. Global Peace Index 2019 list of countries is from: Measuring Peace in a Complex World, Sydney, June 2019. Available from: http://visionofhumanity.org/reports (Consulted March 2020).

|  |  |  |  |  |  | Top high income 2020 |  |  |
| --- | --- | --- | --- | --- | --- | --- | --- | --- |
|  | 2019 GPI |  |  | 2019 GPI |  |  | 2019 GPI |  |
| South Sudan | 3.526 |  | Belize | NA |  | 1 | Luxembourg | 1.071 |
| Somalia | 3.300 |  | Mexico | 2.600 |  | 2 | Norway | 1.536 |
| Central Africa Republic | 3.296 |  | Honduras | 2.341 |  | 3 | Switzerland | 1.059 |
| Congo DR | 3.218 |  | Nicaragua | 2.312 |  | 4 | Qatar | 1.676 |
| Sudan | 2.995 |  | Guatemala | 2.264 |  | 5 | Ireland | 1.390 |
| Niger | 2.898 |  | El Salvador | 2.262 |  | 6 | Denmark | 1.316 |
| Nigeria | 2.898 |  | Panama | 1.804 |  | 7 | Sweden | 1.533 |
| Mali | 2.710 |  | Costa Rica | 1.706 |  | 8 | Australia | 1.071 |
| Cameroon | 2.538 |  | Venezuela | 2.671 |  | 9 | US | 2.401 |
| Chad | 2.522 |  | Colombia | 2.661 |  | 10 | Netherlands | 1.530 |
| Egypt | 2.521 |  | Brazil | 2.271 |  | 11 | Canada | 1.327 |
| Burundi | 2.520 |  | Trinidad | 2.094 |  | 12 | Austria | 1.291 |
| Eritrea | 2.504 |  | Guyana | 2.075 |  | 13 | Japan | 1.369 |
| Zimbabwe | 2.463 |  | Paraguay | 2.055 |  | 14 | Iceland | 1.390 |
| Ethiopia | 2.434 |  | Bolivia | 2.044 |  | 15 | Germany | 1.547 |
| South Africa | 2.399 |  | Peru | 2.016 |  | 16 | Finland | 1.488 |
| Mauritania | 2.333 |  | Argentina | 1.989 |  | 17 | Belgium | 1.533 |
| Republic of Congo | 2.323 |  | Ecuador | 1.980 |  | 18 | United Kingdom | 1.801 |
| Kenya | 2.300 |  | French Guiana | - |  | 19 | France | 1.892 |
| Guinea-Bissau | 2.237 |  | Suriname | - |  | 20 | New Zealand | 1.221 |
| Algeria | 2.219 |  |  |  |  | 21 | Italy | 1.754 |
| Djibouti | 2.207 |  | Afghanistan | 3.574 |  | 22 | Spain | 1.699 |
| Togo | 2.205 |  | Bangladesh | 3.412 |  | 23 | Korea (Republic of) | 1.867 |
| Cote d’Ivoire | 2.203 |  | Bhutan | 3.072 |  | 24 | Greece | 1.933 |
| Uganda | 2.196 |  | India | 2.605 |  | 25 | Czech Republic | 1.375 |
| Burkina Faso | 2.176 |  | Nepal | 2.409 |  |  |  |  |
| Lesotho | 2.167 |  | Pakistan | 2.128 |  |  |  |  |
| Gabon | 2.112 |  | Saudi Arabia | 2.003 |  |  |  |  |
| Mozambique | 2.099 |  | Yemen | 1.506 |  |  |  |  |
| Morocco | 2.070 |  |  |  |  |  |  |  |
| Tunisia | 2.035 |  | Brunei | - |  |  |  |  |
| Rwanda | 2.014 |  | Philippines | 2.516 |  |  |  |  |
| Angola | 2.012 |  | Myanmar | 2.357 |  |  |  |  |
| Benin | 1.986 |  | Thailand | 2.278 |  |  |  |  |
| Eswatini | 1.986 |  | China | 2.217 |  |  |  |  |
| Equatorial Guinea | 1.957 |  | Cambodia | 2.066 |  |  |  |  |
| Guinea | 1.957 |  | Sri Lanka | 1.986 |  |  |  |  |
| Gambia | 1.908 |  | Vietnam | 1.877 |  |  |  |  |
| Namibia | 1.892 |  | Timor-Leste | 1.805 |  |  |  |  |
| Liberia | 1.889 |  | Lao PDR | 1.801 |  |  |  |  |
| Senegal | 1.883 |  | Indonesia | 1.785 |  |  |  |  |
| Tanzania | 1.860 |  | Taiwan | 1.725 |  |  |  |  |
| Sierra Leone | 1.822 |  | Malaysia | 1.529 |  |  |  |  |
| Zambia | 1.805 |  | Japan | 1.369 |  |  |  |  |
| Ghana | 1.796 |  | Singapore | 1.347 |  |  |  |  |
| Malawi | 1.779 |  |  |  |  |  |  |  |
| Botswana | 1.676 |  |  |  |  |  |  |  |
|  |  |  |  |  |  |  |  |  |
| Madagascar | 1.867 |  |  |  |  |  |  |  |
